# Effects of statin therapy on diagnoses of new-onset diabetes and worsening glycaemia in large-scale randomised blinded statin trials: an individual participant data meta-analysis

**DOI:** 10.1016/S2213-8587(24)00040-8

**Published:** 2024-03-27

**Authors:** Cholesterol Treatment Trialists’ (CTT) Collaboration, Writing committee, Christina Reith, David Preiss, Lisa Blackwell, Jonathan Emberson, Enti Spata, Kelly Davies, Heather Halls, Charlie Harper, Lisa Holland, Kate Wilson, Alistair J Roddick, Christopher P Cannon, Robert Clarke, Helen M Colhoun, Paul N Durrington, Shinya Goto, Graham A Hitman, G Kees Hovingh, J Wouter Jukema, Wolfgang Koenig, Ian Marschner, Borislava Mihaylova, Connie Newman, Jeffrey L Probstfield, Paul M Ridker, Marc S Sabatine, Naveed Sattar, Gregory G Schwartz, Luigi Tavazzi, Andrew Tonkin, Stella Trompet, Harvey White, Salim Yusuf, Jane Armitage, Anthony Keech, John Simes, Rory Collins, Colin Baigent, CTT secretariat, Jane Armitage, Colin Baigent, Elizabeth Barnes, Lisa Blackwell, Rory Collins, Kelly Davies, Jonathan Emberson, Jordan Fulcher, Heather Halls, William G Herrington, Lisa Holland, Anthony Keech, Adrienne Kirby, Borislava Mihaylova, Rachel O’Connell, David Preiss, Christina Reith, John Simes, Kate Wilson, CTT collaborating trialists, A to Z trial (phase Z), Michael Blazing, Eugene Braunwald, James de Lemos, Sabina Murphy, Terje R Pedersen, Marc Pfeffer, Harvey White, Stephen Wiviott, AFCAPS/TEXCAPS (AirForce/Texas Coronary Atherosclerosis Prevention Study), Michael Clearfield, John R Downs, Antonio Gotto, Stephen Weis, ALERT (Assessment of Lescol in Renal Transplantation), Bengt Fellström, Hallvard Holdaas, Alan Jardine, Terje R Pedersen, ALLHAT (Antihypertensive and Lipid-Lowering Treatment to Prevent Heart Attack Trial), David Gordon, Barry Davis, Curt Furberg, Richard Grimm, Sara Pressel, Jeffrey L Probstfield, Mahboob Rahman, ALLIANCE (Aggressive Lipid-Lowering Initiation Abates New Cardiac Events), Michael Koren, ASCOT (Anglo-Scandinavian Cardiac Outcomes Trial), Bjorn Dahlöf, Ajay Gupta, Neil Poulter, Peter Sever, Hans Wedel, ASPEN (Atorvastatin Study for the Prevention of Coronary Heart Disease Endpoints in Non-Insulin Dependent Diabetes Mellitus), Robert H Knopp, AURORA (A Study to Evaluate the Use of Rosuvastatin in Subjects On Regular Haemodialysis: an Assessment of Survival and Cardiovascular Events), Stuart Cobbe, Bengt Fellström, Hallvard Holdaas, Alan Jardine, Roland Schmieder, Faiez Zannad, CARDS (Collaborative Atorvastatin Diabetes Study), D John Betteridge, Helen M Colhoun, Paul N Durrington, John Fuller, Graham A Hitman, Andrew Neil, CARE (Cholesterol And Recurrent Events Study), Eugene Braunwald, Barry Davis, C Morton Hawkins, Lemuel Moyé, Marc Pfeffer, Frank Sacks, CORONA (Controlled Rosuvastatin Multinational Trial in Heart Failure), John Kjekshus, Hans Wedel, John Wikstrand, 4D (Die Deutsche Diabetes Dialyse Studie), Christoph Wanner, Vera Krane, GISSI (Gruppo Italiano per lo Studio della Sopravvivenza nell’Infarto miocardico) Heart Failure and Prevention trials, Maria Grazia Franzosi, Roberto Latini, Donata Lucci, Aldo Maggioni, Roberto Marchioli, Enrico B Nicolis, Luigi Tavazzi, Gianni Tognoni, HOPE-3, Jackie Bosch, Eva Lonn, Salim Yusuf, HPS (Heart Protection Study), Jane Armitage, Louise Bowman, Rory Collins, Anthony Keech, Martin Landray, Sarah Parish, Richard Peto, IDEAL (Incremental Decrease in Endpoints through Aggressive Lipid-lowering), John JP Kastelein, Terje R Pedersen, JUPITER (Justification for the Use of Statins in Prevention: an Intervention Trial Evaluating Rosuvastatin), Robert Glynn, Antonio Gotto, John JP Kastelein, Wolfgang Koenig, Jean MacFadyen, LIPID (Long-term Intervention with Pravastatin in Ischaemic Disease), Anthony Keech, Stephen MacMahon, Ian Marschner, Andrew Tonkin, John Shaw, John Simes, LIPS (Lescol Intervention Prevention Study), Patrick W Serruys, Post-CABG (Post-Coronary Artery Bypass Graft Study), Genell Knatterud, PROSPER (Prospective Study of Pravastatin in the Elderly at Risk), Gerard J Blauw, Stuart Cobbe, Ian Ford, Peter Macfarlane, Chris Packard, Naveed Sattar, James Shepherd, Stella Trompet, PROVE-IT (Pravastatin or Atorvastatin Evaluation and Infection Therapy), Eugene Braunwald, Christopher P Cannon, Sabina Murphy, SEARCH (Study of Effectiveness of Additional Reductions in Cholesterol and Homocysteine), Rory Collins, Jane Armitage, Louise Bowman, Richard Bulbulia, Richard Haynes, Sarah Parish, Richard Peto, Peter Sleight, SPARCL (Stroke Prevention by Aggressive Reduction in Cholesterol Levels), Pierre Amarenco, K Michael Welch, 4S (Scandinavian Simvastatin Survival Study), John Kjekshus, Terje R Pedersen, Lars Wilhelmsen, TNT (Treating to New Targets), Philip Barter, Antonio Gotto, John LaRosa, John JP Kastelein, James Shepherd, WOSCOPS (West of Scotland Coronary Prevention Study), Stuart Cobbe, Ian Ford, Sharon Kean, Peter Macfarlane, Chris Packard, Michele Robertson, Naveed Sattar, James Shepherd, Robin Young, Other CTT members, Hiroyuki Arashi, Robert Clarke, Marcus Flather, Shinya Goto, Uri Goldbourt, Jemma Hopewell, G Kees Hovingh, George Kitas, Connie Newman, Marc S Sabatine, Gregory G Schwartz, Liam Smeeth, Jonathan Tobert, John Varigos, Independent oversight committee, Emily Banks, Michael Blastland, Stephen Evans, Robert Temple, Peter Weissberg, Janet Wittes

## Abstract

**Background:**

Previous meta-analyses of summary data from randomised controlled trials have shown that statin therapy increases the risk of diabetes, but less is known about the size or timing of this effect, or who is at greatest risk. We aimed to address these gaps in knowledge through analysis of individual participant data from large, long-term, randomised, double-blind trials of statin therapy.

**Methods:**

We conducted a meta-analysis of individual participant data from randomised controlled trials of statin therapy that participated in the CTT Collaboration. All double-blind randomised controlled trials of statin therapy of at least 2 years’ scheduled duration and with at least 1000 participants were eligible for inclusion in this meta-analysis. All recorded diabetes-related adverse events, treatments, and measures of glycaemia were sought from eligible trials. Meta-analyses assessed the effects of allocation to statin therapy on new-onset diabetes (defined by diabetes-related adverse events, use of new glucose-lowering medications, glucose concentrations, or HbA_1c_ values) and on worsening glycaemia in people with diabetes (defined by complications of glucose control, increased use of glucose-lowering medication, or HbA_1c_ increase of ≥0·5%). Standard inverse-variance-weighted meta-analyses of the effects on these outcomes were conducted according to a prespecified protocol.

**Findings:**

Of the trials participating in the CTT Collaboration, 19 trials compared statin versus placebo (123 940 participants, 25 701 [21%] with diabetes; median follow-up of 4·3 years), and four trials compared more versus less intensive statin therapy (30 724 participants, 5340 [17%] with diabetes, median follow-up of 4·9 years). Compared with placebo, allocation to low-intensity or moderate-intensity statin therapy resulted in a 10% proportional increase in new-onset diabetes (2420 of 39 179 participants assigned to receive a statin [1·3% per year] *vs* 2214 of 39 266 participants assigned to receive placebo [1·2% per year]; rate ratio [RR] 1·10, 95% CI 1·04–1·16), and allocation to high-intensity statin therapy resulted in a 36% proportional increase (1221 of 9935 participants assigned to receive a statin [4·8% per year] *vs* 905 of 9859 participants assigned to receive placebo [3·5% per year]; 1·36, 1·25–1·48). For each trial, the rate of new-onset diabetes among participants allocated to receive placebo depended mostly on the proportion of participants who had at least one follow-up HbA_1c_ measurement; this proportion was much higher in the high-intensity than the low-intensity or moderate-intensity trials. Consequently, the main determinant of the magnitude of the absolute excesses in the two types of trial was the extent of HbA_1c_ measurement rather than the proportional increase in risk associated with statin therapy. In participants without baseline diabetes, mean glucose increased by 0·04 mmol/L with both low-intensity or moderate-intensity (95% CI 0·03–0·05) and high-intensity statins (0·02–0·06), and mean HbA_1c_ increased by 0·06% (0·00–0·12) with low-intensity or moderate-intensity statins and 0·08% (0·07–0·09) with high-intensity statins. Among those with a baseline measure of glycaemia, approximately 62% of new-onset diabetes cases were among participants who were already in the top quarter of the baseline distribution. The relative effects of statin therapy on new-onset diabetes were similar among different types of participants and over time. Among participants with baseline diabetes, the RRs for worsening glycaemia were 1·10 (1·06–1·14) for low-intensity or moderate-intensity statin therapy and 1·24 (1·06–1·44) for high-intensity statin therapy compared with placebo.

**Interpretation:**

Statins cause a moderate dose-dependent increase in new diagnoses of diabetes that is consistent with a small upwards shift in glycaemia, with the majority of new diagnoses of diabetes occurring in people with baseline glycaemic markers that are close to the diagnostic threshold for diabetes. Importantly, however, any theoretical adverse effects of statins on cardiovascular risk that might arise from these small increases in glycaemia (or, indeed, from any other mechanism) are already accounted for in the overall reduction in cardiovascular risk that is seen with statin therapy in these trials. These findings should further inform clinical guidelines regarding clinical management of people taking statin therapy.

## Introduction

Atherosclerotic cardiovascular disease is a leading cause of death worldwide, and LDL cholesterol is a major causal risk factor.^1^ Diabetes substantially increases the risk of atherosclerotic cardiovascular disease.^2^ Randomised controlled trials have shown that prolonged reduction of LDL cholesterol concentrations with a 3-hydroxy-3-methylglutaryl-coenzyme A (HMG Co-A) reductase inhibitor (ie, a statin) reduces the incidence of myocardial infarction and ischaemic stroke by about a quarter for every 1 mmol/L reduction in LDL cholesterol,^3^ with consistent effects in individuals with and without diabetes.^4^

Statins have few confirmed adverse effects,^5^ but meta-analyses of summary data in published reports from large randomised controlled trials of statin therapy indicated that standard statin regimens increased the risk of new-onset diabetes by about 10% compared with placebo or usual care^6^ and that more intensive statin regimens produced a further 10% relative increase in risk.^7^ However, due to the limited information available for these meta-analyses of summary data, assessment of the effects of statin therapy on the risk of developing new diabetes is incomplete. In particular, little is known about which types of people are at particularly high risk of developing diabetes due to a statin, the timing of any excess risk after commencing therapy, or the effects of statin therapy on glycaemic control in people with known diabetes.

To provide insights into these and related questions, we sought individual participant data on all recorded diabetes-related adverse events, treatments for diabetes, and measures of glycaemia recorded within the large, long-term, double-blind, randomised controlled trials of statin therapy that participate in the Cholesterol Treatment Trialists’ (CTT) Collaboration.

## Methods

### Search strategy and selection criteria

Methods were described prospectively in the published CTT Collaboration protocol.^8^ Briefly, we conducted a meta-analysis of individual participant data from randomised controlled trials of statin therapy participating in the CTT Collaboration. Double-blind, randomised controlled trials of statin therapy were eligible for inclusion if there were no protocol-mandated differences between treatment groups other than those created by allocation to receive statin versus placebo or allocation to receive more intensive statin therapy versus less intensive statin therapy; they involved at least 1000 participants; and there was a mean scheduled follow-up of at least 2 years. We requested individual participant data related to all adverse events recorded during the scheduled period of treatment and follow-up. These data included the timing of such events, use of other medications (including glucose-lowering medications), physical measurements, any comorbidities, and laboratory results (including glucose and HbA_1c_ values; appendix p 2).

### Data analysis

We converted data into a common domain-based format on the basis of the Clinical Data Interchange Standards Consortium Study Data Tabulation Model,^9,10^ and all adverse event terms were mapped to the Medical Dictionary for Regulatory Activities, version 20.0 (appendix pp 3–6).^10^ Diabetes-related adverse events were diabetes diagnosis, diabetes-specific complications related to ketosis and glucose control, and any other diabetes-specific complications (appendix pp 3–6). Glucose-lowering drugs were identified by use of a drug dictionary based on Martindale (appendix p 7).^11^ Glucose concentrations were categorised according to fasting status and assumed to be non-fasting when fasting status was unknown. HbA_1c_ values were recorded as percentages rather than mmol/mol because most of the trials were conducted before the introduction of the International Federation of Clinical Chemistry and Laboratory Medicine standard units for HbA_1c_.^12^

Baseline diabetes was defined as a recorded history of diabetes, adverse event of diabetes (appendix pp 3–6) on or before the date of participant assignment to a treatment group, use of glucose-lowering medication (appendix p 7), fasting plasma glucose concentration of 7·0 mmol/L or higher or random plasma glucose of 11·1 mmol/L or higher, or HbA_1c_ value of 6·5% or higher. For participants without baseline diabetes, the outcome of new-onset diabetes was defined as the first record after participant assignment to a treatment group of an adverse event of diabetes, use of glucose-lowering medication, at least two measurements (not necessarily consecutive) of fasting plasma glucose concentration 7·0 mmol/L or higher or random plasma glucose concentration of 11·1 mmol/L or higher, or at least one HbA_1c_ value of 6·5% or higher (based on widely used biochemical thresholds).^13,14^ For participants with baseline diabetes, the outcome of worsening glycaemia was defined as a recording after participant assignment to a treatment group of an adverse event relating to ketosis or complications of glucose control, an HbA_1c_ increase (from baseline) of 0·5% or higher, or escalation of glucose-lowering medication (ie, starting such medication for participants not on medication at baseline, starting insulin for those not on insulin therapy at baseline, or an increase in the number of non-insulin glucose-lowering medications, with or without insulin). Variables for which data were extracted were specified previously.^8^

We calculated the log-rank observed-minus-expected statistic (o – e) and its variance (v) for the first occurrence of each outcome among participants assigned to a treatment group in each trial.^15^ The inverse-variance-weighted average of log of the rate ratio (log RR) across all trials was then calculated as S/V (with variance 1/V, and hence with 95% CI of S/V ±1·96/√V), where S is the sum of (o – e) over all trials and V is the sum of v over all trials. This approach gives nearly identical estimates to the hazard ratio from a trial-stratified Cox regression model. Prespecified subgroup analyses included analyses according to particular baseline participant characteristics, by year of treatment, and for different statin regimens or intensities. Standard χ^2^ tests for heterogeneity (or trend) in the log RR were conducted to assess whether the effect in any given subgroup differed materially from the overall effect seen in all participants.^15^ Exploratory analyses examined the effects of weighting each trial by the trial-specific absolute LDL cholesterol concentration difference at 1 year (as previously described).^3^ Overall RRs are reported with 95% CIs, but all other RRs (eg, in subgroup analyses) are reported with 99% CIs to provide some allowance for multiple comparisons. The effects of allocation to statin therapy on mean glucose concentrations and HbA_1c_ values after assignment to a treatment group were calculated using inverse-variance-weighted meta-analyses.

In addition to the prespecified subgroup analyses, additional post-hoc analyses were done to further explore variation according to baseline levels of glycaemia by dividing participants into quartiles defined hierarchically on the basis of HbA_1c_, fasting glucose concentration (if HbA_1c_ value was not available), or random glucose concentration (if neither HbA_1c_ value or fasting glucose concentration were available). A further post-hoc analysis explored the effect of statin therapy on mean difference in weight subdivided by statin intensity and presence of baseline diabetes.

Results are reported separately for low-intensity or moderate-intensity and high-intensity statin regimens (according to the American Heart Association–American College of Cardiology guideline definition;^16^ appendix p 8). Only two trials^17,18^ allowed for direct assessments of high-intensity statin versus placebo, but indirect assessments of the effects of high-intensity statin therapy were calculated as described previously.^19^

To estimate the average absolute effect of statin therapy on the underlying rate of particular outcomes, we applied the RR (or its lower and upper 95% CIs) to the absolute rate in the appropriate comparator group. We used the summary RRs for all statin regimens in 16 trials^17,18,20–33^ of statin versus placebo to estimate the absolute excess annual rate of new-onset diabetes according to quartiles of baseline glycaemia and a risk score of new-onset diabetes, developed using a Poisson regression model (with the logarithm of follow-up time set as an offset variable) that incorporated univariate predictors of new-onset diabetes (namely baseline age, sex, BMI, triglycerides, estimated glomerular filtration rate [eGFR], HDL cholesterol concentration, and glycaemia; appendix p 28).

All analyses were based on the intention-to-treat principle. Analyses were done using SAS (version 9.4) and R (version 4.1.3). In all trials, participants gave informed consent. Ethics approval for this meta-analysis was subsequently granted by the UK National Health Service Health Research Authority (21/SC/0071).

### Role of the funding source

The funders of the study had no role in study design, data collection, data analysis, data interpretation, or writing of the report.

## Results

Of the trials in the CTT Collaboration, individual participant data were available from 19 eligible double-blind trials^17,18,20–36^ of any statin regimen versus placebo (123 940 participants; median follow-up of 4·3 years), of which 16 trials^17,18,20–33^ (117 437 participants) included participants with and without a history of diabetes, and three trials^34–36^ (6503 participants) recruited only participants with a history of diabetes (table). One trial^20^ (6605 participants) compared a low-intensity statin regimen with placebo, 16 trials^21–36^ (95 890 participants) compared a moderate-intensity statin with placebo, and two trials^17,18^ (21 445 participants) compared a high-intensity statin regimen with placebo. Among all 19 trials, 22 925 (18%) of 123 940 participants had a known history of diabetes at randomisation, and an additional 2776 (2%) participants met our definition of baseline diabetes (appendix p 9).

Individual participant data were also available from four double-blind trials^37–40^ of more versus less intensive statin regimens (30 724 participants; median follow-up of 4·9 years; table). In these four trials, two trials^39,40^ (14 163 participants; median follow-up of 4·1 years) compared high-intensity versus moderate-intensity statin regimens, and two trials^37,38^ (16 561 participants; median follow-up of 5·6 years) compared two moderate-intensity statin regimens. Among all four trials of more versus less intensive statin, 4589 (15%) of 30 724 participants had a known history of diabetes at baseline, and an additional 751 (2%) met our definition of baseline diabetes (appendix p 9).

**Figure 1:**
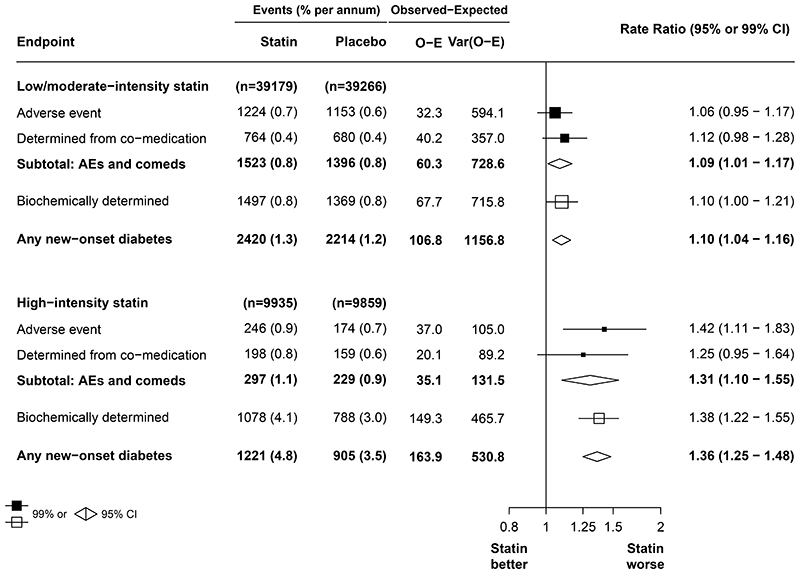
Effect of statin *vs* placebo on new-onset diabetes by statin intensity Test for heterogeneity between low-intensity or moderate-intensity and high-intensity regimens for the outcome of any new-onset diabetes (p<0·0001). Var(o – e) represents the variance of the log-rank observed-minus-expected statistic.

In the 14 trials^20–33^ of low-intensity or moderate-intensity statin versus placebo that included participants without diabetes at baseline, allocation to statin therapy resulted in a 10% relative increase in new-onset diabetes (2420 of 39 179 participants assigned to statin therapy [1·3% per year] *vs* 2214 of 39 266 participants assigned to placebo [1·2% per year]; RR 1·10, 95% CI 1·04–1·16), which corresponded to a mean absolute excess of 0·12% (95% CI 0·04–0·20) during each year of treatment (figure 1). The RRs were similar irrespective of the mode of diagnosis (figure 1; appendix pp 12–15).

The placebo event rate for new-onset diabetes was substantially higher in the two trials of high-intensity statin (905 of 9859 participants assigned to placebo [3·5% per year]) than in the 14 trials of low-intensity or moderate-intensity statins (1·2% per year), and this difference was driven by biochemical diagnosis of diabetes (788 of 9859 participants assigned to placebo [3·0% per year] for high-intensity statins *vs* 1369 of 39 266 participants assigned to placebo [0·8% per year] for low-intensity or moderate-intensity statins; figure 1). Notably, in the high-intensity statin trials, HbA_1c_ was measured at least once after assignment to a treatment group in 14 345 (72%) of 19 794 participants without diabetes at baseline (all of which were in the JUPITER trial^17^) and glucose concentration was measured at least twice after assignment to a treatment group in 9785 (49%) of 19 794 participants without diabetes at baseline, making a biochemical diagnosis possible. By comparison, HbA_1c_ values after assignment to a treatment group were available for just 2434 (3%) of 78 445 participants and glucose concentrations after assignment to a treatment group were available for 29 008 (37%) of 78 445 participants in the low-intensity or moderate-intensity trials. In the two trials^17,18^ of high-intensity statin versus placebo that included participants without baseline diabetes, allocation to statin therapy resulted in a 36% relative increase in new-onset diabetes (1221 of 9935 participants assigned to statin therapy [4·8% per year] *vs* 905 of 9859 participants assigned to placebo [3·5% per year]; RR 1·36, 95% CI 1·25–1·48; figure 1), representing an absolute annual excess of 1·27% (95% CI 0·88–1·69). Although the absolute excess risk of new-onset diabetes varied depending on the method of diagnosis, the RRs were broadly similar (appendix p 16).

Further information on the risks of new-onset diabetes for statin regimens of differing intensity was available from four trials of more versus less intensive statin therapy.^37–40^ Compared with less intensive statin therapy, more intensive statin therapy resulted in a 10% proportional increase in new-onset diabetes (RR 1·10, 95% CI 1·02–1·18), corresponding to an absolute annual excess of 0·22% (95% CI 0·05–0·41; appendix pp 17–18). The RR for high-intensity statin derived indirectly by combining selected trials of more versus less intensive statin and low-intensity or moderate-intensity statin versus placebo was 1·27 (95% CI 1·11–1·44; data not shown), which was similar to the estimate obtained in the direct comparison of high-intensity statin versus placebo (1·36, 1·25–1·48; figure 1).

Overall, at a given level of statin intensity, the relative effects on new-onset diabetes did not vary much in different types of participants (eg, by age, sex, race or ethnicity, history of vascular disease, BMI, eGFR, quartiles of glycaemia, diabetes risk score, and lipid characteristics; appendix pp 19–24), between statins (appendix p 15), or over time (appendix pp 25–26). In particular, the RRs for new-onset diabetes were similar among quartiles of baseline glycaemia and quartiles of baseline-defined risk of new-onset diabetes (appendix pp 19, 21). They were also similar when RRs were weighted for absolute differences in LDL cholesterol at 1 year between trials (low-intensity or moderate-intensity statin versus placebo, RR 1·09, 95% CI 1·03–1·15; high-intensity statin versus placebo, 1·31, 1·21–1·41).

In the trials of statin versus placebo, glucose concentrations were recorded systematically at baseline and follow-up among all people without diabetes in seven trials and HbA_1c_ values were recorded in this way in two trials (appendix p 2). The mean increase in glucose concentration during the treatment period compared with participants assigned to receive placebo was 0·04 mmol/L for both low-intensity or moderate-intensity (95% CI 0·03–0·05) and high-intensity statin therapy (0·02–0·06), and the corresponding increases in HbA_1c_ values were 0·06% (0·00–0·12) for low-intensity or moderate-intensity and 0·08% (0·07–0·09) for high-intensity statin therapy (appendix p 10).

**Figure 2:**
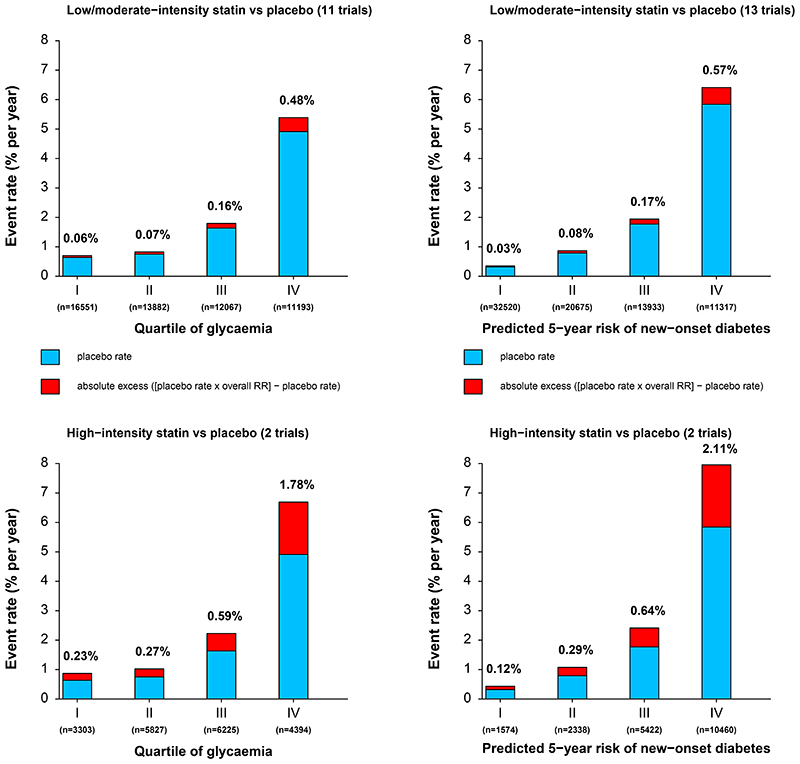
Absolute excess rates of new-onset diabetes in trials of statin versus placebo Rates are shown by quartile of glycaemia (A) and quartile of predicted 5-year risk of new-onset diabetes (B) for low-intensity or moderate-intensity statins and by quartile of glycaemia (C) and quartile of predicted 5-year risk of new-onset diabetes (D) for high-intensity statins. The rate ratio for each group at a specific level of intensity is assumed to be constant. Mean HbA_1c_ for group 1 of glycaemia is 4·72%, for group 2 of glycaemia is 5·51%, for group 3 of glycaemia is 5·80%, and for group 4 of glycaemia is 6·17% for low-intensity or moderate-intensity therapy. Mean HbA_1c_ for group 1 of glycaemia is 5·13%, for group 2 is 5·51%, for group 3 is 5·79%, and for group 4 is 6·14% for high-intensity therapy. Details of the risk score for new-onset diabetes are described in the methods and in the appendix (p 28). Individuals were categorised into four equally sized groups of predicted 5-year risk of new-onset diabetes: <2·9% (group 1), 2·9% to <5·7% (group 2), 5·7% to <11·5% (group 3), and ≥11·5% (group 4).

The annual rate of development of new-onset diabetes in the placebo group was substantially greater in higher versus lower quartiles of baseline glycaemia. Consequently, the majority (ie, approximately 62%) of excess cases of new-onset diabetes occurred among participants in the highest quarter of the baseline glycaemia distribution for both low-intensity or moderate-intensity and high-intensity statin therapy (figure 2). The proportion of excess cases in the top quarter increased only slightly to approximately 67% when baseline age, sex, BMI, triglycerides, eGFR, and HDL cholesterol were added to glycaemia in a diabetes risk score (figure 2).

**Figure 3:**
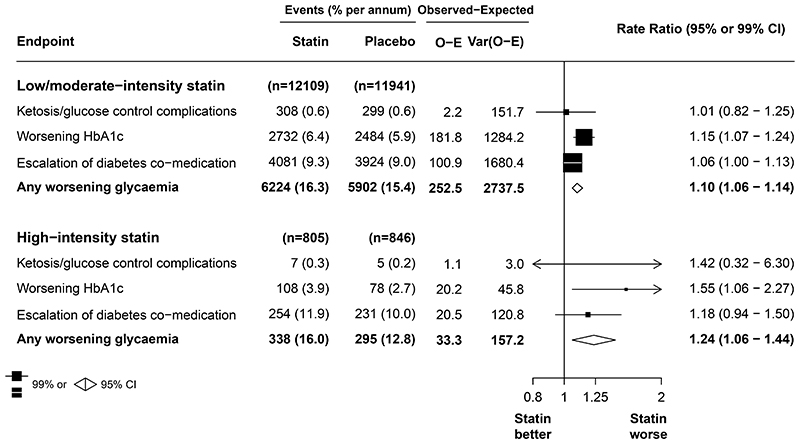
Effect of statin *vs* placebo on worsening glycaemia by statin intensity Test for heterogeneity between low-intensity or moderate-intensity and high-intensity regimens for the outcome of any worsening glycaemia (p=0·15). Var(o – e) represents the variance of the log-rank observed-minus-expected statistic.

Among people with diabetes at baseline, allocation to low-intensity or moderate-intensity statin resulted in a 10% relative increase in worsening glycaemia compared with placebo (6224 of 12 109 participants assigned to statin therapy [16·3% per year] *vs* 5902 of 11 941 participants assigned to placebo [15·4% per year]; RR 1·10 [95% CI 1·06 to 1·14]; absolute annual excess 1·49% [0·87 to 2·13]), and in the high-intensity trials, allocation to this group resulted in a 24% relative increase in worsening glycaemia (338 of 805 participants assigned to statin therapy [16·0% per year] *vs* 295 of 846 participants assigned to placebo [12·8% per year]; 1·24 [1·06 to 1·44]; absolute annual excess 3·02% [0·73 to 5·69]; figure 3). In the trials of low-intensity or moderate-intensity statin versus placebo and the trials of more versus less intensive statin versus placebo, the relative effects on worsening glycaemia were larger in the earlier than later years of follow-up (appendix pp 26–27). The mean increase in glucose concentration during the treatment period compared with participants assigned to receive placebo was 0·12 mmol/L (95% CI 0·04 to 0·21) for low-intensity or moderate-intensity statin therapy and 0·22 mmol/L (–0·02 to 0·45) for high-intensity statin therapy, and the corresponding increases in HbA_1c_ were 0·09% (0·05 to 0·14) for low-intensity or moderate-intensity statin therapy and 0·24% (0·09 to 0·38) for high-intensity statin therapy (appendix p 10).

12 placebo-controlled trials recorded at least one measure of bodyweight in participants without diabetes after assignment to a treatment group. In these participants, the mean baseline weight was 78·14 kg (SD 14·67), and allocation to statin therapy resulted in an increase of 0·16 kg (95% CI 0·08 to 0·24) at 1 year and 0·30 kg (0·22 to 0·37) at the final measurement (appendix p 11) compared with placebo. 11 placebo-controlled trials recorded at least one measure of bodyweight in participants with diabetes after assignment to a treatment group. In these participants, the mean baseline weight was 81·27 kg (SD 14·61), and allocation to statin therapy resulted in an increase of 0·02 kg (–0·10 to 0·14) at 1 year and 0·04 kg (–0·15 to 0·23) at the final measurement compared with placebo.

## Discussion

This meta-analysis advances our understanding of the adverse effects of statin therapy on diabetes. The results show that statin therapy causes a moderate dose-dependent increase in new diagnoses of diabetes, that most of the excess of new-onset diabetes occurs among individuals who are already at high risk of diabetes (ie, their plasma markers of glycaemia are close to the diagnostic threshold for diabetes), and that new-onset diabetes in these individuals is likely to be explained by small statin-induced increases in markers of glycaemia (ie, plasma glucose and HbA_1c_). The relative effects on worsening glycaemic control in people with known diabetes largely mirrored those for new-onset diabetes.

**Figure 4:**
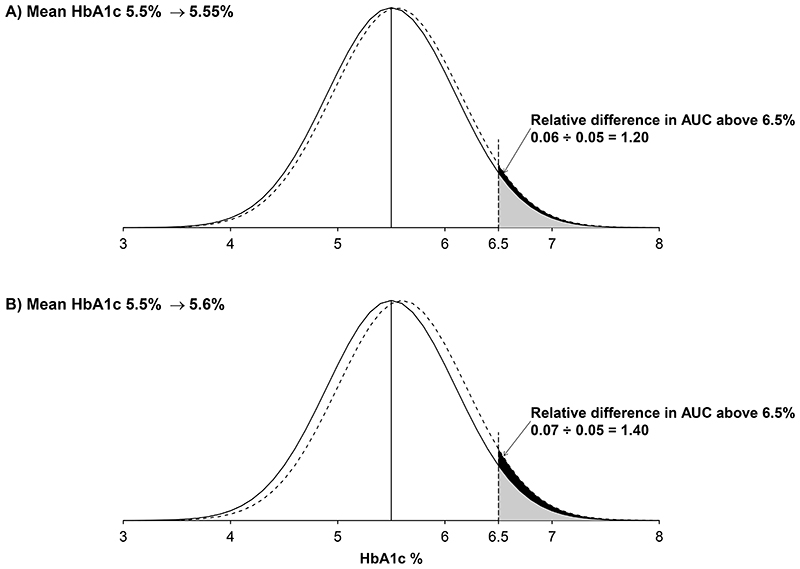
Examples of the effects of population-wide upwards shifts in mean HbA_1c_ Effects of population-wide upwards shifts of 0·05% (A) or 0·10% (B) in mean HbA_1c_ on the proportion above the threshold level of 6·50%. We assumed a normal distribution of HbA_1c_ with a mean of 5·50% (SD 0·60). The SD is taken from the UK Biobank population.^44^ AUC=area under the curve.

The JUPITER trial was the first large randomised trial of statin therapy to report a significant increase in the risk of incident diabetes (270 participants assigned to receive 20 mg rosuvastatin *vs* 216 participants assigned to receive placebo; p=0·01; corresponding to a 25% proportional increase in physician-diagnosed diabetes for participants in the rosuvastatin group).^17^ More recently, the REPRIEVE trial reported a higher rate of incident diabetes in participants assigned to receive 4 mg pitavastatin daily compared with placebo (RR 1·35, 95% CI 1·09–1·66).^41^ Atorvastatin has also been reported to induce a small increase in blood glycaemia within a few months of starting treatment, both in people without diabetes^42^ and in those with diabetes.^43^ Small population-wide shifts in blood glycaemia (of the magnitude seen in our analyses) can have a large relative effect on the proportion of a population exceeding a diagnostic threshold level near the tail of the distribution (figure 4), as evidenced by other drugs that produce small changes in glycaemia but result in moderately large relative changes in the risk of diabetes. For example, in the Diabetes Prevention Program trial, allocation to metformin reduced HbA_1c_ by approximately 0·1% and also reduced the risk of diabetes by 31% compared with placebo,^45^ and in the dal-OUTCOMES trial, which studied dalcetrapib, a reduction in HbA_1c_ of a similar size resulted in approximately 23% reduction in risk compared with placebo.^46^

Overall, there was little availability of data from post-randomisation glycaemic measures among people without known diabetes (appendix p 2). This scarcity was particularly true for HbA_1c_, which was recorded systematically at baseline and at least once during followup among all people without diabetes in only two trials of statin versus placebo (GISSI-HF trial of low-intensity or moderate-intensity statin therapy^31^ [mean baseline HbA_1c_ 5·5%]; JUPITER trial of high-intensity statin therapy^17^ [mean baseline HbA_1c_ 5·7%]; appendix p 9). The paucity of HbA_1c_ data is not surprising because HbA_1c_ did not become a widely recognised diabetes diagnostic marker until 2011,^14^ which was after the inception of all trials included in our analyses. Additionally, it was not always possible to reliably ascertain whether glucose concentration was measured in a fasting or non-fasting state. Given these caveats, to allow for systematic differences in data capture between trials and ensure that the absolute excess rates of new-onset diabetes between trials were comparable, we analysed the excess rates excluding diagnoses made with biochemical measures of glycaemia alone. When this exclusion was made, the RRs overall for low-intensity or moderate-intensity and high-intensity statin therapy were similar to when such biochemical measures were included (figure 1).

In the high-intensity statin trials, the event rate for the development of new-onset diabetes was substantially higher in both the intervention and placebo groups than that seen in the low-intensity or moderate-intensity statin trials. This higher rate was driven by a greater proportion of trial participants in the high-intensity statin trials, particularly in the JUPITER trial, having at least one follow-up HbA_1c_ measurement. Biochemically determined diabetes rates were 3·0% per annum for high-intensity trials and 0·8% for low-intensity or moderate-intensity therapy trials in the placebo groups, whereas rates of diabetes determined by reports of diabetes-related adverse events and use of glucose-lowering medication in the placebo groups for the same groups of trials were similar (figure 1). This finding indicates that, although the relative excesses of new-onset diabetes observed for low-intensity or moderate-intensity statin versus placebo and high-intensity statin versus placebo are likely to be robust and generalisable, the differences in absolute excesses of diagnoses of diabetes between these two groups of trials were determined predominantly by the proportion of trial participants for whom a biochemical diagnosis was made solely through an HbA_1c_ measurement after randomisation. In practice, such measurements might not be obtained routinely in people without diabetes, but it is likely that the rate of diagnosis of diabetes would be higher than it currently is if such a practice was widely adopted.

The RRs for new-onset diabetes did not vary significantly over time. We hypothesise that the reason for this finding is that, in each successive year of follow-up, a new group of people becomes at risk of exceeding the diagnostic threshold for diabetes because of an age-related increase in glycaemia, and those taking a statin will be slightly more likely to do so. For high-intensity statin therapy, the absolute rates were observed to be greater for JUPITER compared with SPARCL, particularly when biochemical measurements of glycaemia were included as a diagnostic criterion (appendix p 16). By contrast, among people with a known diagnosis of diabetes at baseline, the early excess of worsening glycaemia with a statin did not persist in the long term (appendix pp 26–27), perhaps because glycaemic control is typically monitored in such individuals and likely to be managed.

Previous scientific literature has suggested that the increased risk of diabetes caused by statin therapy might be partly due to an increase in bodyweight, which in turn increases diabetes risk.^47^ Data from several trials and meta-analyses have provided an indication of the probable association between bodyweight and diabetes. In the DPP trial, among 3234 individuals without diabetes, lifestyle intervention reduced bodyweight by 5·6 kg and was associated with a 58% (95% CI 48 to 66) reduction in the incidence of type 2 diabetes.^45^ Evidence also exists from meta-analyses of randomised controlled trials of lifestyle interventions for diabetes prevention: in one analysis, compared with usual treatment, a mean bodyweight reduction of 2·45 kg (95% CI –3·56 to –1·33) was associated with a 37% (0·51 to 0·79) reduction in progression to type 2 diabetes at 3 years.^48^ The observed increase in bodyweight due to statin therapy in participants without diabetes in our analyses (ie, 0·30 kg at final measurement; appendix p 11) was much smaller than in these studies. It therefore seems implausible that such a small change in bodyweight would explain more than a small proportion of the observed increase in diagnoses of diabetes due to statin therapy.

A comparison of the cardiovascular benefits and risks of diabetes from statin therapy based on the results of the JUPITER trial^49^ previously concluded that the cardiovascular benefits of rosuvastatin greatly outweighed the risks of new-onset diabetes, despite this trial being conducted in a primary prevention setting among apparently healthy people (without hyperlipidaemia but with increased concentration of CRP on a high-sensitivity CRP test). Notably, vascular benefits of statin therapy represent the net effect of the aggregate effects of statins on blood lipids and glycaemia, such that any theoretical adverse effects of statins on cardiovascular risk that might arise from small increases in glycaemia (or, indeed, from any other mechanism) are already accounted for in the overall reduction in cardiovascular risk that is seen with statin therapy in these trials. Furthermore, the risk of future new major vascular events is significantly greater following major vascular events than following a diagnosis of diabetes.^50,51^ It was not possible to assess clinically significant microvascular complications of diabetes in our analyses both because of the absence of longer-term adverse event data (since development of such complications typically requires many years of exposure to poor glycaemic control) and the absence of any consistent detailed diagnostic information (eg, retinal photographs and measures of microalbuminuria or proteinuria). However, in a meta-analysis of randomised controlled trials comparing less intensive with more intensive glucose control, there was a 20% relative increase in risk of clinically significant renal complications (absolute excess risk 0·4% per year) and a 13% relative increase in risk of clinically significant retinal complications (absolute excess risk 0·2% per year) due to exposure to 0·9% higher HbA_1c_ over 5 years in major diabetes trials,^52^ so the changes induced by a statin are likely to be too small to result in a material change in the risk of microvascular disease in people with diabetes.

Our findings have several implications for clinical practice. First, our findings make clear that the majority of new diagnoses of diabetes resulting from statin therapy will occur among people who are already close to the biochemical diagnostic threshold for diabetes. In our study, approximately 62% of cases of new-onset diabetes attributable to statin therapy occurred among individuals in the top quarter of the glycaemia distribution, and adding other risk factors to glycaemia resulted in only a modest increase (to approximately 67%) in the proportion of cases attributable to statin therapy than for glycaemia alone. Our findings also imply that, since the effect of statin therapy on measures of glycaemia within an individual is small (ie, considerably smaller than the combined variation of within-individual^53^ and laboratory analytical variation^54^), there is likely to be little clinical benefit in measuring glucose concentrations and HbA_1c_ values routinely after starting statin therapy with the aim of making comparisons to values taken before the initiation of a statin. However, people should continue to be screened for diabetes and associated risk factors and have their glycaemic control monitored in accordance with current clinical guidelines.

Although our study emphasises the effects of various statin regimens on the risk of a new diagnosis of diabetes, it does have some limitations. The most important of these limitations is that most of the included trials were not principally designed to test a hypothesis of the effects of statin therapy on diabetes. As aforementioned, one consequence of this was a paucity of data for measures of glycaemia among those without diabetes. Event rates for cases resulting from measurement of fasting plasma glucose might have been overestimated if participants did not fast, although the absolute differences between active and placebo groups would not be materially biased, and exclusion of cases of biochemically determined diabetes did not substantially affect findings. Moreover, cases of diabetes in our analysis were constructed by use of trial data, and we were unable to assess type of diabetes, but we expect that the vast majority of cases in participants of the age included in the trials would have been type 2 diabetes. Very occasionally, glucose-lowering medication might have been used for an indication other than diabetes, and although we were able to count initiation and escalation of diabetes treatment, we were not able to analyse any changes in doses of these medications. The intention-to-treat analyses of the effects of allocation to statin therapy in this meta-analysis preserve the randomised comparisons within each trial, but might of course result in some underestimation of the full effects of taking statin therapy in the long term. Additionally, some data were unavailable for our analyses: data from 218 (8·5%) of 2555 participants in the AURORA trial,^32^ 27 (0·5%) of 4982 participants in the CORONA trial,^30^ and 1088 (6·5%) of 16 714 participants in the JUPITER^17^ trial were not provided because of data privacy concerns. However, it is unlikely that missing data would have affected our main conclusions.

Among people without diabetes, statin therapy produces a dose-dependent increase in the rate of diagnosis of diabetes by inducing a very small increase in glycaemia. People are most at risk of exceeding the diagnostic threshold for diabetes due to statin therapy if their glycaemic control is close to the threshold before treatment. The diabetes-related risks arising from the small changes in glycaemia resulting from statin therapy are greatly outweighed by the benefits of statins on major vascular events when the direct clinical consequences of these outcomes are taken into consideration.

## Supplementary Material

Web appendix

## Figures and Tables

**Table:** Characteristics of the participating trials

	Year of publication of primary results	Number of participants	Treatment comparison	Median follow up, years	Mean LDL cholesterol concentration, mmol/L (SD)	Mean age, years (SD)	Mean BMI, kg/m^2^ (SD)	Mean estimated GFR, mL/min per 1·73 m^2^(SD)	Women, n (%)	White participants, n (%)	Participants with a history of vascular disease, n (%)	Participants with diabetes at baseline, n (%)†
Statin vs placebo (19 trials)	··	123 940	··	4·3	3·5 (0·7)	63 (8)	27·2 (4·1)	69·5 (15·0)	34 533 (28%)	60 152 (81%)*	59 610 (48%)	25 701 (21%)
Low-intensity statin (one trial)	··	6605	··	5·0	3·9 (0·4)	58 (7)	26·9 (3·1)	65·4 (11·6)	997 (15%)	5860 (89%)	0	232 (4%)
AFCAPS/TexCAPS^20^	1998	6605	Lovastatin 20–40 mg/day *vs* placebo	5·0	3·9 (0·4)	58 (7)	26·9 (3·1)	65·4 (11·6)	997 (15%)	5860 (89%)	0	232 (4%)
Moderate-intensity statin (16 trials)	··	95 890	··	4·6	3·6 (0.8)	63 (8)	27·2 (4·2)	69·5 (15·3)	25 254 (26%)	49 877 (80%)*	54 879 (57%)	23 818 (25%)
4S^23^	1994	4444	Simvastatin 20–40 mg/day *vs* placebo	5·4	4·9 (07)	59 (7)	26·0 (3·3)	NA	827 (19%)	NA	4444 (100%)	202 (5%)
WOSCOPS^25^	1995	6595	Pravastatin 40 mg/day *vs* placebo	4·8	5·0 (0.5)	55 (6)	26·0 (3·2)	77·8 (12·4)	0	NA	1066 (16%)	143 (2%)
CARE^26^	1996	4159	Pravastatin 40 mg/day *vs* placebo	4·9	3·6 (0·4)	59 (9)	27·6 (4·4)	67·2 (15·7)	576 (14%)	3851 (93%)	4159 (100%)	667 (16%)
LIPID^27^	1998	9014	Pravastatin 40 mg/day *vs* placebo	5·9	3·9 (0·8)	61 (8)	26·8 (3·8)	70·6 (16·3)	1516 (17%)	NA	9014 (100%)	1077 (12%)
LIPS^22^	2002	1677	Fluvastatin 80 mg/day *vs* placebo	4·0	30·4(0·8)	60 (10)	26·5 (3·3)	67·6 (15·5)	271 (16%)	1650 (98%)	1677 (100%)	204 (12%)
HPS^24^	2002	20 536	Simvastatin 40 mg/day *vs* placebo	5·2	3·4 (0·8)	64 (8)	27·6 (4·4)	72·2 (16·5)	5082 (25%)	19901 (97%)	17386 (85%)	5973 (29%)
PROSPER^28^	2002	5804	Pravastatin 40 mg/day *vs* placebo	3·3	3·8 (0·8)	75 (3)	26·8 (4·2)	56·7 (13·6)	3000 (52%)	NA	2565 (44%)	760 (13%)
ASCOT-LLA^29^	2003	10 240	Atorvastatin 10 mg/day *vs* placebo	3·3	3·4 (0·7)	63 (9)	28·6 (4·6)	68·4 (12·9)	1919 (19%)	9687 (95%)	1684 (16%)	2699 (26%)
ALERT^21^	2003	2102	Fluvastatin 40–80 mg/day *vs* placebo	5·5	4·1 (1·0)	50 (11)	25·8 (4·5)	49·6 (17·0)	715 (34%)	2039 (97%)	409 (19%)	430 (20%)
CARDS^36^	2004	2838	Atorvastatin 10 mg/day *vs* placebo	4·2	2·9 (0·8)	61 (8)	28·8 (3·6)	64·2 (11·3)	909 (32%)	2676 (94%)	106 (4%)	2838 (100%)
4D^35^	2005	1255	Atorvastatin 20 mg/day *vs* placebo	2·7	3·3 (0·8)	66 (8)	27·6 (4·8)	NA	578 (46%)	924 (74%)	1041 (83%)	1255 (100%)
ASPEN^34^	2006	2410	Atorvastatin 10 mg/day *vs* placebo	4·0	2·9 (0·7)	60 (8)	28·9 (3·8)	65·9 (12·8)	811 (34%)	2029 (84%)	747 (31%)	2410 (100%)
CORONA^30^	2007	4982	Rosuvastatin 10 mg/day *vs* placebo	2·7	3·6 (0·9)	72 (7)	26·4 (3·6)	55·4 (15·1)	1175 (24%)	NA	4982 (100%)	1481 (30%)
GISSI-HF^31^	2008	4574	Rosuvastatin 10 mg/day *vs* placebo	3·9	3·1 (0·9)	68 (11)	27·1 (4·5)	66·3 (20·4)	1032 (23%)	4574 (100%)	4574 (100%)	1771 (39%)
AURORA^32^	2009	2555	Rosuvastatin 10 mg/day *vs* placebo	3·9	2·6 (0·9)	64 (9)	24·8 (3·9)	NA	969 (38%)	NA	1025 (40%)	747 (29%)
HOPE-3^33^	2016	12 705	Rosuvastatin 10 mg/day *vs* placebo	5·5	3·3 (0·9)	66 (6)	27·1 (4·7)	79·6 (16·1)	5874 (46%)	2546 (20%)	0	1161 (9%)
High-intensity statin (two trials)	··	21 445	··	2·6	2·9 (0·5)	65 (9)	27·6 (4·0)	70·7 (14·6)	8282 (39%)	4415 (93%)*	4731 (22%)	1651 (8%)
SPARCL^18^	2006	4731	Atorvastatin 80 mg/day *vs* placebo	4·9	3·5 (0·6)	63 (11)	27·9 (5·2)	65·2 (13·8)	1908 (40%)	4415 (93%)	4731 (100%)	909 (19%)
JUPITER^17^	2008	16 714	Rosuvastatin 20 mg/day *vs* placebo	1·9	2·7 (0·5)	65 (8)	27·5 (3·6)	72·3 (14·8)	6374 (38%)	NA	0	742 (4%)
More intensive *vs* less intensive statin (double blind; four trials)	··	30 724	··	4·9	2·5 (0·6)	62 (9)	28·4 (5·1)	72·2 (15·6)	5965 (19%)	28865 (94%)	30724 (100%)	5340 (17%)
Comparison of moderate-intensity regimens (two trials)	··	16 561	··	5·6	2·4(0·6)	63 (9)	28·0(4·3)	74·8 (16·8)	3152 (19%)	15 679 (95%)	16 561 (100%)	2339 (14%)
A to Z^37^	2004	4497	Simvastatin 40 mg/day then 80 mg/day *vs* placebo then simvastatin 20 mg/day	2·0	2·1 (0·5)	60 (11)	27·6 (4·8)	68·4 (16·0)	1100 (24%)	3825 (85%)	4497 (100%)	1059 (24%)
SEARCH^38^	2010	12 064	Simvastatin 80 mg/day *vs* 20 mg/day	7·0	2·5 (0·6)	64 (9)	28·1 (4·1)	77·2 (17·1)	2052 (17%)	11 854 (98%)	12 064 (100%)	1280 (11%)
Comparison of high-intensity *vs* moderate-intensity regimens (two trials)	··	14 163	··	4·1	2·5 (0·6)	60 (10)	29·0 (6·0)	69·1 (14·3)	2813 (20%)	13 186 (93%)	14 163 (100%)	3001 (21%)
PROVE-IT^39^	2004	4162	Atorvastatin 80 mg/day *vs* pravastatin 40 mg/day	2·1	2·6 (0·7)	58 (11)	29·5 (5·7)	78·8 (18·7)	911 (22%)	3776 (91%)	4162 (100%)	1034 (25%)
TNT^40^	2005	10 001	Atorvastatin 80 mg/day *vs* 10 mg/day	5·0	2·5 (0·5)	61 (9)	28·8 (6·1)	65·0 (12·4)	1902 (19%)	9410 (94%)	10 001 (100%)	1967 (20%)
All trials	··	154 664	··	4·4	3·3 (0·7)	63 (8)	27·5 (4·3)	70·1 (15·1)	40 498 (26%)	89 017 (85%)*	90 334 (58%)	31041 (20%)

All trials randomised in a 1:1 allocation. Some participants in the AURORA (n=218), CORONA (n=27), and JUPITER (n=1088) trials withdrew consent for use of their data after the trial, and hence data from these participants is excluded. The ASCOT-LLA trial excludes 65 participants for whom data were not available due to protocol violations, and so are not included in the number of participants or percentages shown. 4D=Die Deutsche Diabetes Dialyse Studie. 4S=Scandinavian Simvastatin Survival Study. AFCAPS/TexCAPS=Air Force/Texas Coronary Atherosclerosis Prevention Study. ALERT=Assessment of Lescol in Renal Transplantation. ASCOT-LLA=Anglo-Scandinavian Cardiac Outcomes Trial-Lipid Lowering Arm. ASPEN=Atorvastatin Study for Prevention of Coronary Heart Disease Endpoints in Non-Insulin-Dependent Diabetes Mellitus. A to Z=Aggrastat to Zocor. AURORA=A Study to Evaluate the Use of Rosuvastatin in Subjects on Regular Hemodialysis: An Assessment of Survival and Cardiovascular Events. CARDS=Collaborative Atorvastatin Diabetes Study. CARE=Cholesterol And Recurrent Events. CORONA=Controlled Rosuvastatin Multinational Trial in Heart Failure. GISSI-HF=Gruppo Italiano per lo Studio della Sopravvivenza nell’Insufficienza cardiaca. HOPE-3=Heart Outcomes Prevention Evaluation-3 trial. HPS=Heart Protection Study. JUPITER=Justification for the Use of Statins in Prevention: an Intervention Trial Evaluating Rosuvastatin. LIPID=Long-term Intervention with Pravastatin in Ischaemic Disease. LIPS=Lescol Intervention Prevention Study. NA=not available. PROSPER=PROspective Study of Pravastatin in the Elderly at Risk. PROVE-IT=Pravastatin or Atorvastatin Evaluation and Infection Therapy. SEARCH=Study of the Effectiveness of Additional Reductions in Cholesterol and Homocysteine. SPARCL=Stroke Prevention by Aggressive Reduction in Cholesterol Levels. TNT=Treating to New Targets. WOSCOPS=West of Scotland Coronary Prevention Study.

* Percentages were calculated after excluding the seven trials where information on race and ethnicity was not provided (the relevant denominators are therefore 73 832 for all trials of statin vs placebo, 62 496 for trials of moderate-intensity statin therapy vs placebo, 4731 for trials of high-intensity statin therapy vs placebo, and 104 556 for all trials).

† Baseline diabetes is defined as participants with a history of diabetes plus those retrospectively defined as having diabetes at baseline on the basis of adverse events, glucose-lowering medication, or glucose or HbA1c measurements at the time of assignment to a treatment group.

## Data Availability

Individual participant data from each contributing trial have been provided to the Cholesterol Treatment Trialists’ Collaboration on the understanding that they would be used only for the purpose of the Cholesterol Treatment Trialists’ meta-analyses and would not be released to others. Requests for such data should be made directly to the data custodians of each trial. The Cholesterol Treatment Trialists’ data policy can be found at https://www.cttcollaboration.org/.

## Research in context

### Evidence before this study

We searched Medline and the Cochrane Central Register of Controlled Trials for randomised trials and meta-analyses published between Jan 1, 1990, and April 1, 2022, that specifically assessed the effects of statin regimens on new-onset diabetes and worsening glycaemia. For example, to identify meta-analyses in Medline, we used the *BMJ* systematic review search strategy in combination with (“statin.mp.” or “exp Hydroxymethylglutaryl-CoA Reductase Inhibitors/”) and (“exp Diabetes Mellitus, Type 1/” or “diabet*.mp” or “exp Diabetes Mellitus/”). Meta-analyses published up until April, 2022, have used summary data from randomised controlled trials to assess the effects of statin therapy on new-onset diabetes. These analyses suggested that statin therapy increases the likelihood of new-onset diabetes being diagnosed, with more intensive statin therapy leading to larger increases. However, they had insufficient detail to investigate these findings in depth, including which individuals were at particular risk, when the effect emerged and its persistence, the effects of different statin regimens, and the effects on glycaemic control in individuals with diabetes.

### Added value of this study

Obtaining individual participant data on all recorded diabetes-related adverse events and treatments, along with serial glycaemia measures, from large, long-term, blinded, randomised controlled trials has allowed the effect of statin therapy on the development of new-onset diabetes and worsening glycaemia to be assessed more comprehensively than has previously been possible with summary level data. Low-intensity or moderate-intensity regimens resulted in a 10% relative increase in new-onset diabetes compared with placebo, and high-intensity statin regimens resulted in a 36% relative increase. These increases persisted when biochemically determined diagnoses of diabetes were excluded. The rate ratios were consistent with a small increase in glycaemia due to statin therapy. These effects were widely generalisable to the different types of participants studied and persisted while treatment continued. The absolute excesses for new-onset diabetes were highest among those individuals in whom measures of glycaemia were already close to the diagnostic threshold for diabetes. Within each trial, the main determinant of the magnitude of the absolute excess was the proportion of trial participants having at least one follow-up HbA_1c_ measurement rather than the proportional increase in risk associated with statin therapy. Any theoretical adverse effects of statins on cardiovascular risk that might arise from these small increases in glycaemia (or, indeed, from any other mechanism) are already accounted for in the overall reduction in cardiovascular risk that is seen with statin therapy in these trials. Our analyses strongly suggest that the absolute benefits of statin therapy greatly outweigh any excess risks of diabetes associated with the small increase in glycaemia they induce.

### Implications of all the available evidence

Statin therapy produces a small increase in glycaemia, which translates into a moderate increase in the rate at which individuals are diagnosed with new-onset diabetes (or worsening glycaemic control among those with diabetes). The mean changes in glycaemia are small, and the evidence of the beneficial effects on major vascular events provides reassurance about the net benefits of using statin therapy in individuals who are at increased risk of developing diabetes or have already developed it.
