# Supplementary material for: Effects of statin therapy on diagnoses of new-onset diabetes and worsening glycaemia in large-scale randomised blinded statin trials: an individual participant data meta-analysis
Source: Lancet Diabetes Endocrinol. Author manuscript; Available in PMC 2024 May 16. (PMC7615958; doi:10.1016/S2213-8587(24)00040-8)
Supplement: Web appendix [file EMS194978-supplement-Web_appendix.pdf]

Individual patient data meta-analysis of new-onset diabetes mellitus and worsening glycaemia in large-scale randomised blinded statin trials

Online Web appendix, Table of Contents

Webtables		Page
1	Summary of diabetes-related data collection in included trials	2
2	Definition of diabetes outcomes	3
3	Classification of glucose-lowering medication	7
4	Intensity of statin therapy	8
5	Descriptive statistics on glycaemia among people with or without diabetes at baseline	9
6	Mean difference from baseline to average follow up in glucose and HbA1c subdivided by statin intensity and presence of baseline diabetes	10
7	Effect of statin vs placebo on mean difference in weight subdivided by statin intensity and presence of baseline diabetes	11

Webfigures		
1a	Effect of statin vs placebo on NEW-ONSET DIABETES (ADVERSE EVENT DETERMINED) among participants with no diabetes at baseline*, subdivided by statin intensity and trial	12
1b	Effect of statin vs placebo on NEW-ONSET DIABETES (CO-MEDICATION DETERMINED) among participants with no diabetes at baseline*, subdivided by statin intensity and trial	13
1c	Effect of statin vs placebo on NEW-ONSET DIABETES (BIOCHEMICALLY DETERMINED) among participants with no diabetes at baseline*, subdivided by statin intensity and trial	14
1d	Effect of statin vs placebo on NEW-ONSET DIABETES, subdivided by statin intensity and trial	15
2	Effect of high-intensity statin vs placebo on NEW-ONSET DIABETES using different diagnosis criteria	16
3	Effect of more vs less intensive statin on NEW-ONSET DIABETES, subdivided by statin intensity and trial	17
4	Effect of more vs less intensive statin on NEW-ONSET DIABETES and WORSENING GLYCAEMIA	18
5	Effect of low/moderate-intensity statin vs placebo on NEW-ONSET DIABETES, subdivided by participants’ characteristics	19
6	Effect of low/moderate-intensity statin vs placebo on NEW-ONSET DIABETES, subdivided by participants' lipid characteristics	20
7	Effect of high-intensity statin vs placebo on NEW-ONSET DIABETES, subdivided by participants' characteristics	21
8	Effect of high-intensity statin vs placebo on NEW-ONSET DIABETES, subdivided by participants' lipid characteristics	22
9	Effect of more vs less intensive statin on NEW-ONSET DIABETES, subdivided by participants' characteristics	23
10	Effect of more vs less intensive statin on NEW-ONSET DIABETES, subdivided by participants' lipid characteristics	24
11	Effect of statin vs placebo on NEW-ONSET DIABETES, subdivided by duration of treatment and statin intensity	25
12	Effect of more vs less intensive statin on NEW-ONSET DIABETES and WORSENING GLYCAEMIA, subdivided by duration of treatment	26
13	Effect of statin vs placebo on WORSENING GLYCAEMIA, subdivided by duration of treatment and statin intensity	27

Statistical appendix		
	Stratifying participants into baseline risk groups of new-onset diabetes	28

Webtable 1: Summary of diabetes-related data collection in included trials

Study	Timing of routine FU visits (months)	Type of event data principally collected	Blood glucose values recorded at baseline	Blood glucose values routinely recorded during FU	HbA1c values routinely recorded at baseline	HbA1c values routinely recorded during FU	Co-medication (all or DM specific) routinely recorded at baseline	Co-medication (all or DM specific) routinely recorded at follow up
**Statin vs Placebo**								
Low-intensity statin therapy								
AFCAPS/ TexCAPS	every 1.5 to 12, 15, 18 then every 6 to 60	All AEs	†Y	N	N	N	Y	N
Moderate-intensity statin therapy								
ALERT	1.5, every 6 to 72	All AEs	†Y	N	N	N	Y	Every visit
LIPS	1.5, 6 then ~every 6	All AEs	N	N	N	N	N	N
4S	every 1.5 to 18, every 6 to 48	All AEs	†Y	N (final FU fasting glucose planned but trial stopped early)	N	N	N	N
HPS	4, 8, 12, every 6 until 60	SAEs + selected AEs	N	N	¶Y	¶Y	Y	Every visit
WOSCOPS	Every 3 to final	All AEs	†Y	Every 3 months in first year; 6 monthly after year 1 (additional measurement possible if abnormal value)	N	N	Y	Every visit
CARE	1.5, every 3 until 72	All AEs	†Y	†3, 6, 12, every 12 until 72	N (unless routine glucose ≥12.2 mmol/L)	N (unless routine glucose ≥12.2 mmol/L)	Y	Every visit
LIPID	3, 6, 9, 12, annually to 72	SAEs	†Y	N	N	N	Y	Every visit
PROSPER	every 3 to final	All AEs	†Y	†At 12 then every 12	N	N	Y	Every visit
4D	1, 6, every 6 to 48	All AEs	†Y	1, 6, 12, every 6 to 48	†Y	†Every visit	Y	Every visit
CARDS	1, 2, 3, 6, every 6 to 48	All AEs	†Y	†12, every 12 to 48	†Y	†12, every 12 to 48	Y	Every visit
ASCOT-LLA	1.5, 3, 6, every 6 until 66/final	All AEs	†Y	†6, 12, 24, 36, 48, 60, 66/final	N	N	Y	Every visit
ASPEN	1, 2, 3, 6, every 6m to 48	All AEs	†Y	Every visit	†Y	†12, 24, 36, 48	Y	Every visit
CORONA	1.5, 3, every 3 to 51, final	All AEs	N	N	N	N	Y	Every visit
GISSI-HF	1, 3, 6, every 6 to 60	SAEs + selected AEs	†Y	†1, 3, 6, 12, annually to 60	†Y	†12, annually to 60	N	N
AURORA	3, 6, every 6 to 42	All AEs	†Y	†Final visit	N	N	Y	Every visit
HOPE-3	1.5, 6, every 6 to 96	SAEs + selected AEs	†Y	N (unless new diagnosis of diabetes)	N	N (unless new diagnosis of diabetes)	Y	24, last visit
High-intensity statin therapy								
SPARCL	1, 3, 6, every 6 to 78	All AEs	†Y	§Y (frequency NK)	NK	NK	Y	Every visit
JUPITER	3, 6, every 6 to 36, Close out	All AEs	†Y	†24, 36	†Y	†24, 36	Y	Every visit
**More intensive vs less intensive statin (double blind)**								
**Comparison of moderate-intensity regimens**								
A to Z	1, 4, every 4 to 24	SAEs + selected AEs	†Y	†Every visit	N	N	Y	Every visit
SEARCH	2, 4, 8, 12, every 6 until 84	SAEs + selected AEs	N	N	*Y	N	Y	Every visit
**Comparison of moderate-intensity regimens**								
TNT	3, 6, 9, 12, every 6 to 72	All AEs	†Y	†12, every 12 to 72	†Y	*12, every 12 to 72	Y	Every visit
PROVE-IT	0.5, 1, 4, every 4 to 28	All AEs	†Y	†1, 4, 8, 16, final visit	†Y	†1, 4, 8, 16, final	Y	Every visit

This is the planned data collection timetable but not all data was available and/or able to be utilized (e.g. because only presented as summary level data). Time is in months.
†Measured in all patients; *Measured only in participants with known/suspected diabetes; ¶Measured in all those reporting diabetes at baseline but only in a random sample once during follow-up; §Not known if measured in all patients or a subset; Y=Yes; N=No/none; NK=Not known

Webtable 2: Definition of diabetes outcomes

Outcome	MedDRA term*	Lower Level Terms associated with Preferred Term
**Diabetes diagnosis**		
	Acquired lipoatrophic diabetes	Acquired lipoatrophic diabetes; Lawrence-Seip syndrome.
	Diabetes complicating pregnancy	Diabetes complicating pregnancy; Diabetes complicating the puerperium.
	Diabetes mellitus	Diabetes; Diabetes mellitus; Diabetes mellitus aggravated; Diabetes mellitus exacerbated; Diabetes mellitus NOS; Diabetes mellitus precipitated; Diabetes mellitus progression; Diabetes mellitus reactivated; Diabetes mellitus without mention of complication; Diabetes reactivated; Diabetes steroid-induced; Diabetic; Post transplant diabetes mellitus; Worsening of diabetes.
	Diabetes mellitus malnutrition-related	Diabetes mellitus malnutrition-related; J type diabetes; Jamaica type diabetes mellitus; Tropical diabetes; Z type diabetes.
	Diabetes with hyperosmolarity	Diabetes with hyperosmolarity.
	Fulminant type 1 diabetes mellitus	Fulminant type I diabetes mellitus; Fulminant type 1 diabetes mellitus.
	Gestational diabetes	Diabetes in pregnancy; Diabetes mellitus gestational; Diabetes mellitus, antepartum; Diabetes mellitus, postpartum; Gestational diabetes; Gestational diabetes mellitus.
	Insulin resistant diabetes	Insulin resistant diabetes.
	Insulin-requiring type 2 diabetes mellitus	Insulin-requiring type II diabetes mellitus; Insulin-requiring type 2 diabetes mellitus.
	Latent autoimmune diabetes in adults	Latent autoimmune diabetes in adults; Slowly progressive insulin dependent diabetes; Type 1.5 diabetes mellitus.
	Monogenic diabetes	Maturity-onset diabetes of the young; Monogenic diabetes.
	Pancreatogenous diabetes	Pancreatogenous diabetes.
	Type 1 diabetes mellitus	Diabetes mellitus insulin-dependent; Diabetes mellitus juvenile onset; IDDM; Insulin dependent diabetic; Insulin-dependent diabetes mellitus; Juvenile diabetes; Ketosis-prone diabetes mellitus; Type 1 diabetes mellitus; Type I diabetes mellitus; Type I diabetes mellitus without mention of complication.
	Type 2 diabetes mellitus	Diabetes mellitus maturity onset; Diabetes mellitus non-insulin-dependent; Maturity onset diabetes; NIDDM; Non-insulin-dependent diabetes mellitus; Type 2 diabetes mellitus; Type II diabetes mellitus; Type II diabetes mellitus without mention of complication; Type II non-obese diabetic; Type II obese diabetic.
	Type 3 diabetes mellitus	Type 3 diabetes mellitus; Type III diabetes mellitus.
	Diabetes mellitus (incl subtypes)*	
**Diabetic-specific complications related to ketosis and glucose control**		
	Diabetes mellitus inadequate control	Brittle diabetes; Diabetes brittle; Diabetes mellitus inadequate control; Diabetes mellitus loss of control; Diabetes mellitus poor control; Diabetic control impaired; Loss of control of blood sugar; Loss of control of diabetes; Type I Diabetes mellitus inadequate control; Type II Diabetes mellitus inadequate control.
	Diabetic ketoacidosis	Acidosis diabetic; Diabetes mellitus with ketoacidosis; Diabetes with ketoacidosis; Diabetic acidosis; Diabetic ketoacidosis; Diabetic ketosis; Ketoacidosis (diabetic);

Outcome	MedDRA term*	Lower Level Terms associated with Preferred Term
		Type I diabetes mellitus with ketoacidosis; Type II diabetes mellitus with ketoacidosis.
	Diabetic metabolic decompensation	Decompensated diabetes; Diabetic metabolic decompensation.
	Diabetic coma	Coma diabetic; Diabetes with coma; Diabetic coma; Diabetic coma NOS.
	Diabetic hyperglycaemic coma	Coma hyperglycaemic; Coma hyperglycemic; Diabetic hyperglycaemic coma; Diabetic hyperglycemic coma.
	Diabetic hyperosmolar coma	Diabetes with hyperosmolar coma; Diabetic hyperosmolar coma; Hyperosmolar (non-ketotic) coma; Hyperosmolar non-ketotic diabetic coma; Nonketotic hyperglycaemic-hyperosmolar coma; Nonketotic hyperglycemic-hyperosmolar coma; Type I diabetes mellitus with hyperosmolar coma; Type II diabetes mellitus with hyperosmolar coma.
	Diabetic ketoacidotic hyperglycaemic coma	Diabetic ketoacidotic hyperglycaemic coma; Diabetic ketoacidotic hyperglycemic coma.
	Hyperglycaemic hyperosmolar nonketotic syndrome	Diabetic hyperosmolar non-ketoacidosis; Hyperglycaemic hyperosmolar nonketotic syndrome; Hyperglycemic hyperosmolar nonketotic syndrome; Hyperosmolar hyperglycaemic state; Hyperosmolar hyperglycaemic syndrome; Hyperosmolar hyperglycemic state; Hyperosmolar hyperglycemic syndrome.
**Cardiovascular diabetic complications**		
	Diabetic arteritis	Diabetic arteritis.
	Diabetic cardiomyopathy	Diabetic cardiomyopathy.
	Diabetic macroangiopathy	Diabetic arteriosclerosis; Diabetic macroangiopathy.
	Diabetic microangiopathy	Diabetic microangiopathy; Small vessel disease of diabetes mellitus.
	Diabetic vascular disorder	Diabetes with peripheral circulatory disorders; Diabetic peripheral angiopathy; Diabetic peripheral vascular disease; Diabetic vascular disorder; Diabetic vascular disorder NOS; Type I diabetes mellitus with peripheral circulatory disorders; Type II diabetes mellitus with peripheral circulatory disorders; Type II diabetes peripheral angiopathy.
**Renal diabetic complications**		
	Diabetic cystopathy	Diabetic cystopathy.
	Diabetic end stage renal disease	Diabetic end stage renal disease.
	Diabetic nephropathy	Diabetes with renal manifestations; Diabetic nephropathy; Diabetic nephropathy NOS; Diabetic renal disease; Type I diabetes mellitus with renal manifestations; Type II diabetes mellitus with renal manifestations.
**Neurological diabetic complications**		
	Acute painful neuropathy of rapid glycaemic control	Acute painful neuropathy of rapid glycaemic control; Acute painful neuropathy of rapid glycemic control; Insulin neuritis.
	Diabetic amyotrophy	Diabetic amyotrophy.
	Diabetic autonomic neuropathy	Diabetic autonomic neuropathy; Diabetic peripheral autonomic neuropathy.
	Diabetic encephalopathy	Diabetic encephalopathy.
	Diabetic mononeuropathy	Diabetic mononeuropathy.
	Diabetic neuropathic ulcer	Diabetic neuropathic ulcer.

Outcome	MedDRA term*	Lower Level Terms associated with Preferred Term
	Diabetic neuropathy	Diabetes with neurological manifestations; Diabetic neuropathy; Diabetic peripheral neuropathic pain; Diabetic peripheral neuropathy; Diabetic polyneuropathy; Polyneuropathy in diabetes; Type I Diabetes mellitus with neurological manifestations; Type II Diabetes mellitus with neurological manifestations.
**Ophthalmic diabetic complications**		
	Cataract diabetic	Cataract diabetic; Diabetic cataract.
	Diabetic blindness	Diabetic blindness.
	Diabetic eye disease	Diabetes with ophthalmic manifestations; Diabetic eye disease; Diabetic eye disease NOS; Type I diabetes mellitus with ophthalmic manifestations; Type II diabetes mellitus with ophthalmic manifestations.
	Diabetic glaucoma	Diabetic glaucoma.
	Diabetic keratopathy	Diabetic keratopathy.
	Diabetic ophthalmoplegia	Diabetic ophthalmoplegia.
	Diabetic retinal oedema	Diabetic macular edema; Diabetic macular oedema; Diabetic retinal edema; Diabetic retinal oedema.
	Diabetic retinopathy	Background diabetic retinopathy; Background retinopathy, unspecified; Diabetic macular retinopathy; Diabetic neovascularisation; Diabetic neovascularization; Diabetic retinal angiopathy; Diabetic retinal microaneurysms; Diabetic retinopathy; Non-proliferative diabetic retinopathy; Preproliferative diabetic retinopathy; Proliferative diabetic retinopathy; Retinopathy background; Retinopathy diabetic; Simple retinopathy.
	Diabetic uveitis	Diabetic uveitis.
**Dermal diabetic complications**		
	Diabetic bullosis	Bullosis diabeticorum; Diabetic blister; Diabetic bullosis.
	Diabetic cheiroarthropathy	Diabetic cheiroarthropathy; Diabetic cheiropathy; Diabetic hand syndrome.
	Diabetic dermopathy	Diabetic dermopathy.
	Diabetic foot	Diabetic foot; Diabetic foot ulcer.
	Diabetic foot infection	Diabetic foot infection.
	Diabetic gangrene	Diabetic gangrene.
	Diabetic ulcer	Diabetic ulcer; Diabetic ulcer NOS.
	Necrobiosis lipoidica diabeticorum	Necrobiosis lipoidica; Necrobiosis lipoidica diabeticorum.
**Gastrointestinal diabetic complications**		
	Diabetic enteropathy	Diabetic enteropathy.
	Diabetic gastroenteropathy	Diabetic gastroenteropathy.
	Diabetic gastroparesis	Diabetic gastroparesis.
	Diabetic gastropathy	Diabetic gastropathy.
**Other diabetes-specific complications/terms**		
	Diabetic arthropathy	Diabetic arthropathy.
	Diabetic complication	Diabetes with unspecified complication; Diabetes with unspecified complications; Diabetic complication; Diabetic complication NOS; Diabetic triopathy.
	Diabetic dyslipidaemia	Diabetic dyslipidaemia; Diabetic dyslipidemia.
	Diabetic endorgan damage	Diabetic endorgan damage.
	Diabetic foetopathy	Diabetic fetopathy; Diabetic foetopathy.
	Diabetic hepatopathy	Diabetic hepatopathy.
	Diabetic mastopathy	Diabetic mastopathy.
	Mauriac syndrome	Mauriac syndrome.

Outcome	MedDRA term*	Lower Level Terms associated with Preferred Term
	Diabetes mellitus management	Diabetes mellitus management; Glycaemia control; Glycemia control.
	Diabetic complications*	

*All terms are MedDRA Preferred Terms apart from those with an asterisk which indicates a Higher Level Term (HLT) or Higher Level Group Term (HLGT). Note that HLTs and HLGTs are only used where a Preferred Term (PT) or Lower Level Term (LLT) was not available for any given trial.

Webtable 3: Classification of glucose-lowering medication

CTT categories*†	Category name
A10A	insulin
A10B	non-insulin glucose lowering agent
A10BA	biguanide
A10BB	sulfonylurea
A10BC	sulfonamide
A10BF	alpha glucosidase inhibitor
A10BG	thiazolidinedione
A10BH	DPP4 inhibitor
A10BJ	GLP1 receptor agonist
A10BK	SGLT2 inhibitor
A10BX	meglitinide

*these categories are based on Martindale's drug reference list

†combination drugs were assigned relevant categories

Webtable 4: Intensity of statin therapy †

Type	Daily dosage in mg, by intensity (LDL-C reduction)		
	Low (<30%)	Moderate (30% to 50%)	High (>50%)
Lovastatin	20	40 to 80	NA
Simvastatin	10	20 to 80‡	NA
Pravastatin	10 to 20	40 to 80	NA
Fluvastatin	20 to 40	2X40 or 80	NA
Atorvastatin	NA	10 to 20	40 to 80
Rosuvastatin	NA	5 to 10	20 to 40
Pitavastatin	NA	1 to 4	NA

† From 2018 AHA/ACC/AACVPR/AAPA/ABC/ACPM/ADA/AGS/APhA/ASPC/NLA/PCNA Guideline on the Management of Blood Cholesterol; A Report of the American College of Cardiology/American Heart Association Task Force on Clinical Practice Guidelines; Table 3; J Am Coll Cardiol. 2019;73(24):e285-e350.

‡ Consistent with the above guideline, we classified simvastatin 80mg as ‘moderate intensity’ based on estimates of the median reduction in LDL-C from the VOYAGER metaanalysis (Eur Heart J Cardiovasc Pharmacother. 2016; 2: 212-7).

Webtable 5: Descriptive statistics on glycaemia among people with or without diabetes at baseline

Trial	Medical history of diabetes (%)	Additionally identified diabetes (%) *	All baseline diabetes (%) *	All patients Glucose (mmol/L)	All patients HbA1c (%)	Patients with diabetes at baseline ** Glucose (mmol/L)	Patients with diabetes at baseline ** HbA1c (%)	Patients without diabetes at baseline ** Glucose (mmol/L)	Patients without diabetes at baseline ** HbA1c (%)
**Statin vs placebo**									
Low-intensity statin									
AFCAPS/TexCAPS	155 (2%)	77 (1%)	232 (4%)	5.0 (0.8)	NA	7.0 (1.4)	NA	4.9 (0.6)	NA
**Subtotal 1 trials**	**155 (2%)**	**77 (1%)**	**232 (4%)**	**5.0 (0.8)**	**NA**	**7.0 (1.4)**		**4.9 (0.6)**	
Moderate-intensity statin									
ALERT	396 (19%)	34 (2%)	430 (20%)	5.2 (2.8)	NA	8.3 (5.0)	NA	4.4 (0.7)	NA
LIPS	202 (12%)	2 (<1%)	204 (12%)	NA	NA	NA	NA	NA	NA
4S	202 (5%)	0	202 (5%)	NA	NA	NA	NA	NA	NA
HPS	5963 (29%)	10 (<1%)	5973 (29%)	NA	7.1 (2.4)	NA	7.1 (2.4)	NA	4.1 (0.7)
WOSCOPS	77 (1%)	66 (1%)	143 (2%)	4.8 (0.7)	NA	7.4 (1.5)	NA	4.7 (0.5)	NA
CARE	586 (14%)	81 (2%)	667 (16%)	5.4 (1.8)	10.6 (2.5)	8.1 (3.0)	10.8 (2.4)	4.9 (0.7)	5.7 (0.3)
LIPID	782 (9%)	295 (3%)	1077 (12%)	5.7 (1.5)	NA	8.4 (2.7)	NA	5.3 (0.6)	NA
PROSPER	623 (11%)	137 (2%)	760 (13%)	5.5 (1.4)	NA	8.1 (2.3)	NA	5.1 (0.6)	NA
4D	1255 (100%)	NA	1255 (100%)	8.7 (3.6)	6.7 (1.3)	8.7 (3.6)	6.7 (1.3)	NA	NA
CARDS	2838 (100%)	NA	2838 (100%)	9.9 (3.2)	7.8 (1.4)	9.9 (3.2)	7.8 (1.4)	NA	NA
ASCOT-LLA	2540 (25%)	159 (2%)	2699 (26%)	6.2 (2.1)	NA	8.5 (2.7)	NA	5.3 (0.6)	NA
ASPEN	2410 (100%)	NA	2410 (100%)	9.3 (3.1)	7.5 (1.3)	9.3 (3.1)	7.5 (1.3)	NA	NA
CORONA	1473 (30%)	8 (<1%)	1481 (30%)	NA	NA	NA	NA	NA	NA
GISSI-HF	1196 (26%)	575 (13%)	1771 (39%)	6.4 (2.5)	6.2 (1.4)	8.2 (3.1)	7.2 (1.5)	5.3 (0.7)	5.5 (0.6)
AURORA	658 (26%)	89 (3%)	747 (29%)	5.6 (2.0)	NA	7.3 (3.0)	NA	4.9 (0.7)	NA
HOPE-3	731 (6%)	430 (3%)	1161 (9%)	5.5 (1.2)	NA	8.0 (2.3)	8.9 (2.9)	5.3 (0.7)	NA
**Subtotal 16 trials**	**21932 (23%)**	**1886 (2%)**	**23818 (25%)**	**6.0 (1.8)**	**7.5 (2.1)**	**8.7 (3.0)**	**7.5 (1.9)**	**5.1 (0.6)**	**4.6 (0.6)**
High-intensity statin									
SPARCL	794 (17%)	115 (2%)	909 (19%)	5.9 (1.8)	NA	8.3 (2.9)	NA	5.3 (0.6)	NA
JUPITER	44 (<1%)	698 (4%)	742 (4%)	5.3 (0.6)	5.7 (0.4)	5.9 (0.8)	6.7 (0.7)	5.2 (0.6)	5.7 (0.4)
**Subtotal 2 trials**	**838 (4%)**	**813 (4%)**	**1651 (8%)**	**5.4 (0.9)**	**5.7 (0.4)**	**7.2 (2.0)**	**6.7 (0.7)**	**5.2 (0.6)**	**5.7 (0.4)**
**Subtotal 19 trials**	**22925 (18%)**	**2776 (2%)**	**25701 (21%)**	**5.8 (1.5)**	**6.9 (1.6)**	**8.5 (2.9)**	**7.5 (1.9)**	**5.1 (0.6)**	**5.1 (0.5)**
**More intensive vs less intensive statin (double blind)**									
Comparison of moderate-intensity regimens									
A to Z	1059 (24%)	0	1059 (24%)	6.9 (2.9)	NA	10.1 (4.0)	NA	5.9 (1.4)	NA
SEARCH	1267 (11%)	13 (<1%)	1280 (11%)	NA	7.4 (1.6)	NA	7.4 (1.6)	NA	5.4 (0.6)
**Subtotal 2 trials**	**2326 (14%)**	**13 (0%)**	**2339 (14%)**	**6.9 (2.9)**	**7.4 (1.6)**	**10.1 (4.0)**	**7.4 (1.6)**	**5.9 (1.4)**	**5.4 (0.6)**
Comparison of high vs moderate-intensity regimens									
TNT	1501 (15%)	466 (5%)	1967 (20%)	6.0 (1.7)	6.5 (1.3)	8.3 (2.5)	7.3 (1.2)	5.4 (0.6)	5.6 (0.4)
PROVE-IT	762 (18%)	272 (7%)	1034 (25%)	6.3 (2.4)	5.7 (1.2)	8.8 (3.2)	7.0 (1.6)	5.3 (0.9)	5.3 (0.4)
**Subtotal 2 trials**	**2263 (16%)**	**738 (5%)**	**3001 (21%)**	**6.1 (1.9)**	**6.3 (1.3)**	**8.5 (2.7)**	**7.2 (1.3)**	**5.4 (0.7)**	**5.5 (0.4)**
**Subtotal 4 trials**	**4589 (15%)**	**751 (2%)**	**5340 (17%)**	**6.3 (2.1)**	**6.8 (1.4)**	**8.9 (3.1)**	**7.3 (1.4)**	**5.5 (0.9)**	**5.5 (0.5)**
**Subtotal: All trials**	**27514 (18%)**	**3527 (2%)**	**31041 (20%)**	**5.9 (1.6)**	**6.9 (1.5)**	**8.6 (2.9)**	**7.5 (1.8)**	**5.2 (0.7)**	**5.2 (0.5)**

See table 1 for definitions
* Additional DM include those pts retrospectively defined as having DM at baseline (who did not have a history of diabetes at randomisation) from glucose/HbA1c measurements, comedications and AEs on day of randomisation.
** Baseline DM defined as pts with a history of diabetes plus those pts retrospectively defined as having DM at baseline (who did not have a history of diabetes at randomisation) from glucose/HbA1c measurements, comedications and AE's on day of randomisation

Webtable 6: Mean difference from baseline to average follow up in glucose and HbA1c subdivided by statin intensity and presence of baseline diabetes

Glucose

			Statin		Placebo		
	Number of trials	Baseline Mean (SD) Glucose	n	Mean (SE)	n	Mean (SE)	Mean diff (95%CI)
**Patients with no diabetes**							
**Low/Moderate intensity vs placebo**	9	5.08 (0.62)	17961	0.28 (0.01)	17810	0.24 (0.00)	0.04 (0.03 to 0.05)
**High intensity vs placebo**	2	5.25 (0.61)	8933	0.17 (0.01)	8840	0.13 (0.01)	0.04 (0.02 to 0.06)
**Patients with diabetes**							
**Low/Moderate intensity vs placebo**	12	8.83 (2.97)	6521	0.23 (0.03)	6391	0.10 (0.03)	0.12 (0.04 to 0.21)
**High intensity vs placebo**	2	7.20 (1.93)	725	0.55 (0.08)	754	0.33 (0.08)	0.22 (-0.02 to 0.45)

HbA1c

			Statin		Placebo		
	Number of trials	Baseline Mean (SD) HbA1c	n	Mean (SE)	n	Mean (SE)	Mean diff (95%CI)
**Patients with no diabetes**							
**Low/Moderate intensity vs placebo**	1	5.52 (0.61)	911	0.23 (0.02)	977	0.17 (0.02)	0.06 (-0.00 to 0.12)
**High intensity vs placebo**	1	5.66 (0.35)	7174	0.29 (0.00)	7075	0.22 (0.00)	0.08 (0.07 to 0.09)
**Patients with diabetes**							
**Low/Moderate intensity vs placebo**	6	7.41 (1.49)	4250	0.26 (0.02)	4165	0.17 (0.02)	0.09 (0.05 to 0.14)
**High intensity vs placebo**	1	6.73 (0.68)	308	0.28 (0.04)	340	0.04 (0.06)	0.24 (0.09 to 0.38)

Note: Glucose measured in mmol/L. HbA1c measured in %.

Webtable 7: Effect of statin vs placebo on mean difference in weight subdivided by statin intensity and presence of baseline diabetes

		Mean difference (95% CI)	
	Baseline mean (SD) weight (kg)	Baseline to year 1 measurement (kg)	Baseline to Final measurement (kg)
**Participants with no diabetes**			
**Low/moderate statin intensity**			
ALERT	76.77 (15.05)	0.35 (-0.02 to 0.71)	0.52 (-0.10 to 1.13)
LIPS	76.41 (11.42)	-0.19 (-0.71 to 0.33)	0.19 (-0.39 to 0.77)
HPS	78.78 (13.30)	n/a	0.13 (-0.10 to 0.35)
WOSCOPS	76.95 (10.73)	0.14 (0.01 to 0.27)	0.32 (0.08 to 0.56)
LIPID	77.88 (12.57)	0.19 (0.01 to 0.37)	0.42 (0.15 to 0.69)
PROSPER	72.57 (13.16)	n/a	0.44 (0.16 to 0.72)
ASCOT-LLA	83.86 (15.00)	n/a	0.29 (0.04 to 0.54)
CORONA	77.50 (14.40)	n/a	0.30 (-0.07 to 0.67)
GISSI-HF	74.70 (14.47)	0.58 (0.34 to 0.82)	0.90 (0.43 to 1.37)
HOPE-3	72.31 (15.36)	n/a	0.12 (-0.07 to 0.32)
**Subtotal**	**77.01 (13.66)**	**0.21 (0.12 to 0.30)**	**0.29 (0.20 to 0.38)**
**Participants with no diabetes**			
**High statin intensity**			
SPARCL	76.94 (14.62)	-0.01 (-0.19 to 0.17)	-0.15 (-0.54 to 0.23)
JUPITER	82.00 (17.91)	n/a	0.40 (0.23 to 0.57)
**Subtotal**	**81.12 (17.34)**	**-0.01 (-0.19 to 0.17)**	**0.31 (0.16 to 0.46)**
**Subtotal: All participants with no diabetes**	**78.14 (14.67)**	**0.16 (0.08 to 0.24)**	**0.30 (0.22 to 0.37)**
**Participants with diabetes**			
**Low/moderate statin intensity**			
ALERT	75.58 (15.76)	-0.11 (-0.96 to 0.75)	-0.42 (-1.62 to 0.78)
LIPS	77.75 (11.44)	-1.16 (-2.59 to 0.27)	-0.21 (-1.87 to 1.45)
HPS	82.16 (15.26)	n/a	-0.03 (-0.41 to 0.36)
WOSCOPS	82.58 (13.63)	-0.37 (-1.18 to 0.44)	1.11 (-0.45 to 2.67)
LIPID	82.37 (14.32)	0.21 (-0.31 to 0.73)	0.38 (-0.47 to 1.22)
DDDD	77.46 (14.96)	-0.07 (-0.52 to 0.38)	-0.17 (-1.01 to 0.67)
CARDS	83.58 (13.01)	-0.00 (-0.20 to 0.19)	0.01 (-0.40 to 0.43)
ASPEN	85.13 (14.30)	0.22 (-0.02 to 0.46)	0.22 (-0.28 to 0.72)
GISSI-HF	77.79 (14.97)	0.14 (-0.19 to 0.48)	0.34 (-0.27 to 0.95)
HOPE-3	75.15 (15.79)	n/a	-0.39 (-0.98 to 0.20)
**Subtotal**	**81.31 (14.56)**	**0.05 (-0.08 to 0.17)**	**0.04 (-0.15 to 0.24)**
**Participants with diabetes**			
**High statin intensity**			
SPARCL	80.64 (15.58)	-0.34 (-0.78 to 0.10)	-0.18 (-1.05 to 0.70)
**Subtotal**	**80.64 (15.58)**	**-0.34 (-0.78 to 0.10)**	**-0.18 (-1.05 to 0.70)**
**Subtotal: All participants with diabetes**	**81.27 (14.61)**	**0.02 (-0.10 to 0.14)**	**0.04 (-0.15 to 0.23)**

Weighting by inverse variance method. Tabular data supplied by D. Preiss for JUPITER, PROSPER, ASCOT-LLA and CORONA (only for those patients with no history of diabetes) based on previously published analyses (Swerdlow DI et al, Lancet. 2015;385:351-61). Results for these trials use the definition of history of diabetes as described in that manuscript.

Webfigure 1a: Effect of statin vs placebo on NEW−ONSET DIABETES (ADVERSE EVENT DETERMINED) among participants with no diabetes at baseline*, subdivided by statin intensity and trial

Trial	Dose	Events (% per annum) Statin (n=49114)	Events (% per annum) Placebo (n=49125)	Observed−Expected O−E	Observed−Expected Var(O−E)	Rate Ratio (95% or 99% CI)
**Low/moderate−intensity statin**						
**Lovastatin**						
AFCAPS/TexCAPS	20−40mg	62 (0.4)	61 (0.4)	0.2	30.7	1.01 (0.63 − 1.60)
**Subtotal**		**62 (0.4)**	**61 (0.4)**	**0.2**	**30.7**	**1.01 (0.71 − 1.43)**
**Fluvastatin**						
ALERT	40−80mg	22 (0.5)	31 (0.7)	−4.4	13.2	0.72 (0.35 − 1.46)
LIPS	80mg	4 (0.1)	9 (0.3)	−2.4	3.2	0.48 (0.11 − 1.98)
**Subtotal**		**26 (0.4)**	**40 (0.6)**	**−6.8**	**16.5**	**0.66 (0.41 − 1.07)**
**Simvastatin**						
4S	20−40mg	51 (0.5)	45 (0.4)	2.8	24.0	1.13 (0.67 − 1.90)
HPS	40mg	223 (0.6)	207 (0.6)	7.3	107.5	1.07 (0.83 − 1.37)
**Subtotal**		**274 (0.6)**	**252 (0.5)**	**10.1**	**131.5**	**1.08 (0.91 − 1.28)**
**Pravastatin**						
WOSCOPS	40mg	30 (0.2)	26 (0.2)	1.8	14.0	1.14 (0.57 − 2.27)
CARE	40mg	32 (0.4)	29 (0.3)	1.3	15.2	1.09 (0.56 − 2.10)
LIPID	40mg	123 (0.6)	129 (0.6)	−3.7	63.0	0.94 (0.68 − 1.30)
PROSPER	40mg	70 (0.9)	56 (0.7)	6.8	31.5	1.24 (0.79 − 1.97)
**Subtotal**		**255 (0.5)**	**240 (0.4)**	**6.3**	**123.7**	**1.05 (0.88 − 1.25)**
**Atorvastatin**						
ASCOT−LLA	10mg	282 (2.4)	254 (2.2)	12.9	133.9	1.10 (0.88 − 1.38)
**Subtotal**		**282 (2.4)**	**254 (2.2)**	**12.9**	**133.9**	**1.10 (0.93 − 1.30)**
**Rosuvastatin**						
CORONA	10mg	105 (2.4)	85 (2.0)	10.1	47.5	1.24 (0.85 − 1.80)
GISSI−HF	10mg	1 (<0.1)	1 (<0.1)	0.0	0.5	
AURORA	10mg	8 (0.3)	15 (0.5)	−3.2	5.7	0.57 (0.20 − 1.68)
HOPE−3	10mg	211 (0.7)	205 (0.6)	2.7	104.0	1.03 (0.80 − 1.32)
**Subtotal**		**325 (0.7)**	**306 (0.7)**	**9.7**	**157.7**	**1.06 (0.91 − 1.24)**
**Any low/moderate−intensity**		**1224 (0.7)**	**1153 (0.6)**	**32.3**	**594.1**	**1.06 (0.97 − 1.14)**
**High−intensity statin**						
**Atorvastatin**						
SPARCL	80mg	85 (1.0)	52 (0.6)	17.1	34.2	1.65 (1.06 − 2.56)
**Subtotal**		**85 (1.0)**	**52 (0.6)**	**17.1**	**34.2**	**1.65 (1.18 − 2.30)**
**Rosuvastatin**						
JUPITER	20mg	161 (0.9)	122 (0.7)	19.9	70.7	1.32 (0.98 − 1.80)
**Subtotal**		**161 (0.9)**	**122 (0.7)**	**19.9**	**70.7**	**1.32 (1.05 − 1.67)**
**Any high−intensity**		**246 (0.9)**	**174 (0.7)**	**37.0**	**105.0**	**1.42 (1.17 − 1.72)**

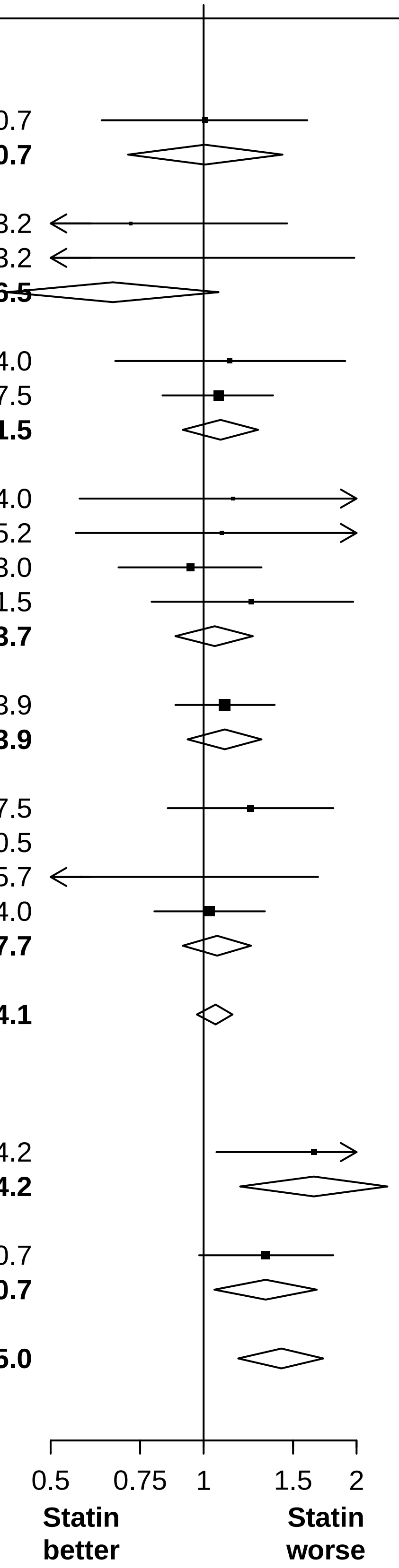

■ 99% or ◇ 95% CI

Test for heterogeneity between 6 statins in the low/moderate−intensity analysis: χ^2_5 =4.0, p=0.56

Test for heterogeneity between 2 statins in the high−intensity trials analysis: χ^2_1 =1.1, p=0.29

Webfigure 1b: Effect of statin vs placebo on NEW−ONSET DIABETES (CO−MEDICATION DETERMINED) among participants with no diabetes at baseline*, subdivided by statin intensity and trial

Trial	Dose	Events (% per annum): Statin (n=49114)	Events (% per annum): Placebo (n=49125)	Observed−Expected: O−E	Observed−Expected: Var(O−E)	Rate Ratio (95% or 99% CI)
**Low/moderate−intensity statin**						
**Lovastatin**						
AFCAPS/TexCAPS	20−40mg	−	−	−	−	
**Subtotal**		**−**	**−**	**−**	**−**	
**Fluvastatin**						
ALERT	40−80mg	23 (0.5)	22 (0.5)	0.7	11.2	1.07 (0.50 − 2.30)
LIPS	80mg	0 (0.0)	2 (<0.1)	−1.0	0.5	
**Subtotal**		**23 (0.3)**	**24 (0.3)**	**−0.2**	**11.7**	**0.98 (0.55 − 1.74)**
**Simvastatin**						
4S	20−40mg	−	−	−	−	
HPS	40mg	242 (0.7)	207 (0.6)	16.3	108.3	1.16 (0.91 − 1.49)
**Subtotal**		**242 (0.5)**	**207 (0.4)**	**16.3**	**108.3**	**1.16 (0.96 − 1.40)**
**Pravastatin**						
WOSCOPS	40mg	17 (0.1)	17 (0.1)	−0.1	8.5	0.99 (0.41 − 2.40)
CARE	40mg	−	−	−	−	
LIPID	40mg	81 (0.4)	66 (0.3)	6.8	36.7	1.20 (0.79 − 1.84)
PROSPER	40mg	27 (0.3)	18 (0.2)	4.4	11.2	1.48 (0.69 − 3.18)
**Subtotal**		**125 (0.2)**	**101 (0.2)**	**11.2**	**56.5**	**1.22 (0.94 − 1.58)**
**Atorvastatin**						
ASCOT−LLA	10mg	50 (0.4)	41 (0.3)	4.2	22.7	1.20 (0.70 − 2.07)
**Subtotal**		**50 (0.4)**	**41 (0.3)**	**4.2**	**22.7**	**1.20 (0.80 − 1.82)**
**Rosuvastatin**						
CORONA	10mg	83 (1.9)	70 (1.6)	6.3	38.2	1.18 (0.78 − 1.79)
GISSI−HF	10mg	−	−	−	−	
AURORA	10mg	43 (1.4)	50 (1.6)	−2.2	23.2	0.91 (0.53 − 1.55)
HOPE−3	10mg	198 (0.6)	187 (0.6)	4.6	96.2	1.05 (0.81 − 1.36)
**Subtotal**		**324 (0.7)**	**307 (0.7)**	**8.7**	**157.7**	**1.06 (0.90 − 1.24)**
**Any low/moderate−intensity**		**764 (0.4)**	**680 (0.4)**	**40.2**	**357.0**	**1.12 (1.01 − 1.24)**
**High−intensity statin**						
**Atorvastatin**						
SPARCL	80mg	85 (1.0)	61 (0.7)	12.4	36.5	1.41 (0.92 − 2.15)
**Subtotal**		**85 (1.0)**	**61 (0.7)**	**12.4**	**36.5**	**1.41 (1.02 − 1.94)**
**Rosuvastatin**						
JUPITER	20mg	113 (0.7)	98 (0.6)	7.6	52.7	1.16 (0.81 − 1.65)
**Subtotal**		**113 (0.7)**	**98 (0.6)**	**7.6**	**52.7**	**1.16 (0.88 − 1.51)**
**Any high−intensity**		**198 (0.8)**	**159 (0.6)**	**20.1**	**89.2**	**1.25 (1.02 − 1.54)**

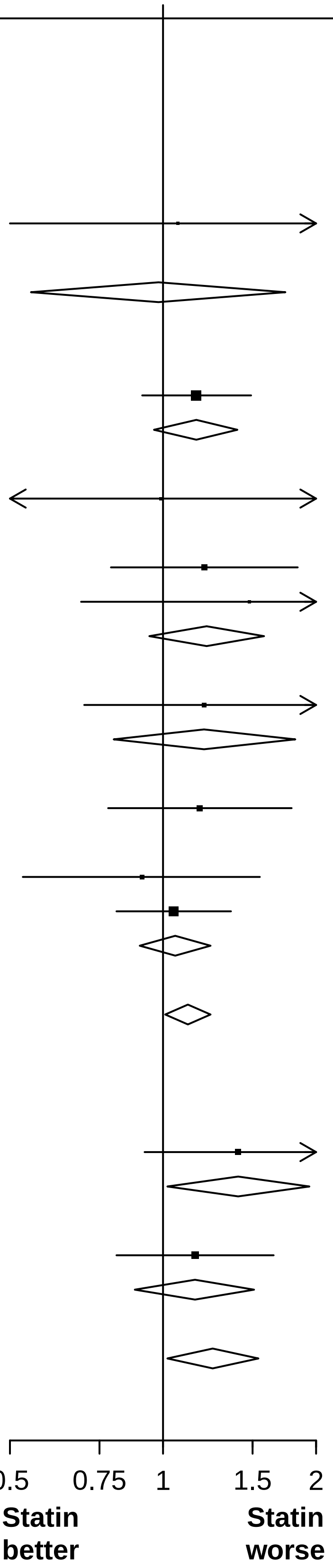

■ 99% or ◇ 95% CI

Test for heterogeneity between 6 statins in the low/moderate−intensity analysis: χ^2_4 =1.4, p=0.84

Test for heterogeneity between 2 statins in the high−intensity trials analysis: χ^2_1 =0.8, p=0.36

Webfigure 1c: Effect of statin vs placebo on NEW−ONSET DIABETES (BIOCHEMICALLY DETERMINED) among participants with no diabetes at baseline*, subdivided by statin intensity and trial

Trial	Dose	Events (% per annum) Statin (n=49114)	Events (% per annum) Placebo (n=49125)	Observed−Expected O−E	Observed−Expected Var(O−E)	Rate Ratio (95% or 99% CI)
**Low/moderate−intensity statin**						
**Lovastatin**						
AFCAPS/TexCAPS	20−40mg	169 (1.1)	130 (0.8)	19.0	74.7	1.29 (0.96 − 1.74)
**Subtotal**		**169 (1.1)**	**130 (0.8)**	**19.0**	**74.7**	**1.29 (1.03 − 1.62)**
**Fluvastatin**						
ALERT	40−80mg	−	−	−	−	
LIPS	80mg	−	−	−	−	
**Subtotal**		**−**	**−**	**−**	**−**	
**Simvastatin**						
4S	20−40mg	−	−	−	−	
HPS	40mg	−	−	−	−	
**Subtotal**		**−**	**−**	**−**	**−**	
**Pravastatin**						
WOSCOPS	40mg	107 (0.7)	126 (0.8)	−10.3	58.2	0.84 (0.60 − 1.17)
CARE	40mg	193 (2.3)	157 (1.9)	16.7	87.4	1.21 (0.92 − 1.59)
LIPID	40mg	160 (0.7)	149 (0.7)	2.6	77.2	1.03 (0.77 − 1.39)
PROSPER	40mg	114 (1.4)	79 (1.0)	17.2	48.2	1.43 (0.99 − 2.07)
**Subtotal**		**574 (1.1)**	**511 (0.9)**	**26.2**	**271.0**	**1.10 (0.98 − 1.24)**
**Atorvastatin**						
ASCOT−LLA	10mg	251 (2.1)	232 (1.9)	8.6	120.6	1.07 (0.85 − 1.36)
**Subtotal**		**251 (2.1)**	**232 (1.9)**	**8.6**	**120.6**	**1.07 (0.90 − 1.28)**
**Rosuvastatin**						
CORONA	10mg	−	−	−	−	
GISSI−HF	10mg	303 (7.0)	299 (6.5)	11.7	150.1	1.08 (0.88 − 1.33)
AURORA	10mg	35 (1.1)	37 (1.1)	−0.1	18.0	1.00 (0.54 − 1.83)
HOPE−3	10mg	165 (0.5)	160 (0.5)	2.2	81.2	1.03 (0.77 − 1.37)
**Subtotal**		**503 (1.2)**	**496 (1.1)**	**13.9**	**249.3**	**1.06 (0.93 − 1.20)**
**Any low/moderate−intensity**		**1497 (0.8)**	**1369 (0.8)**	**67.7**	**715.8**	**1.10 (1.02 − 1.18)**
**High−intensity statin**						
**Atorvastatin**						
SPARCL	80mg	88 (1.0)	70 (0.8)	9.3	39.5	1.26 (0.84 − 1.90)
**Subtotal**		**88 (1.0)**	**70 (0.8)**	**9.3**	**39.5**	**1.26 (0.93 − 1.73)**
**Rosuvastatin**						
JUPITER	20mg	990 (5.8)	718 (4.2)	140.0	426.2	1.39 (1.23 − 1.57)
**Subtotal**		**990 (5.8)**	**718 (4.2)**	**140.0**	**426.2**	**1.39 (1.26 − 1.53)**
**Any high−intensity**		**1078 (4.1)**	**788 (3.0)**	**149.3**	**465.7**	**1.38 (1.26 − 1.51)**

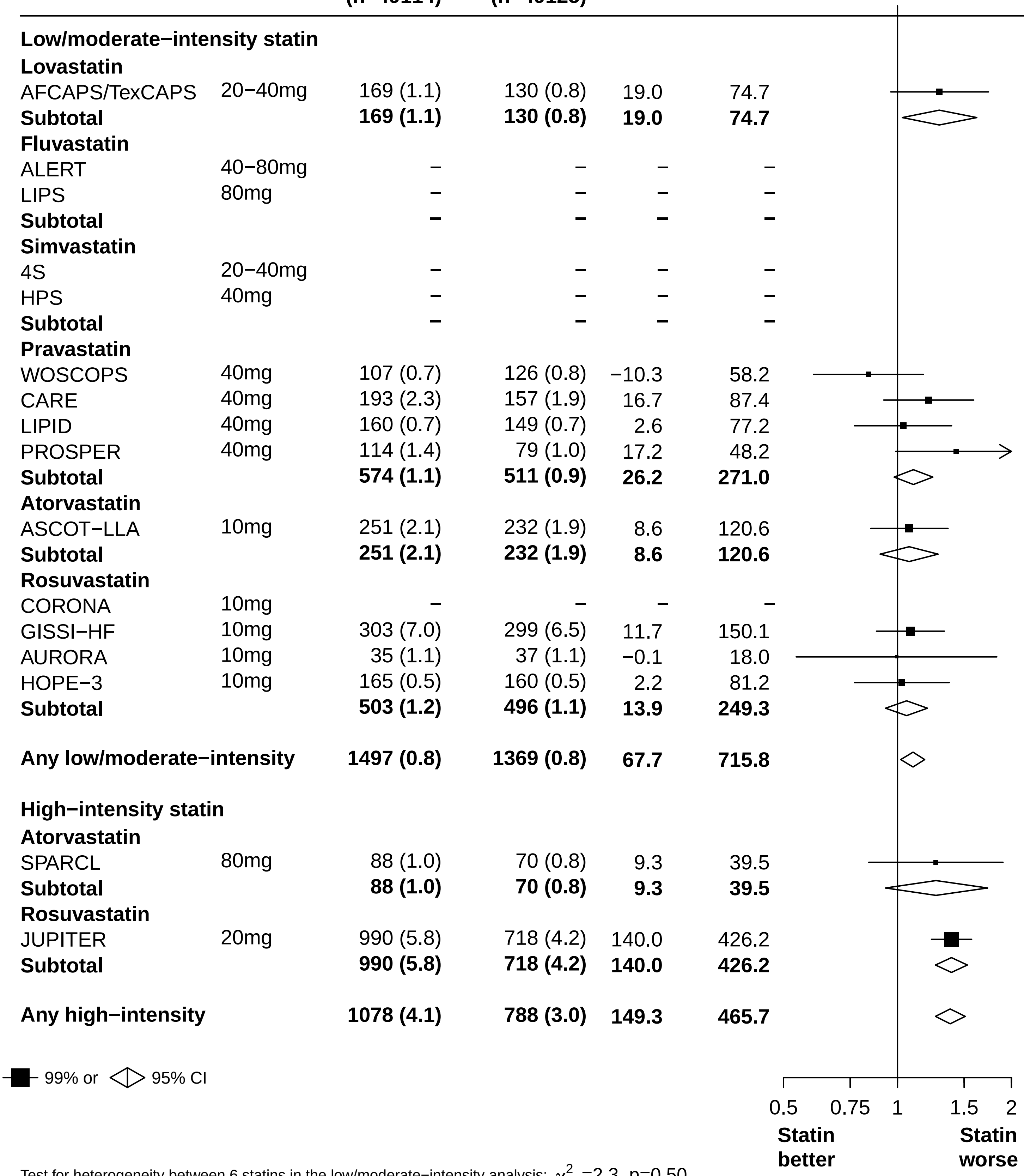

Test for heterogeneity between 6 statins in the low/moderate−intensity analysis: χ^2_3 =2.3, p=0.50

Test for heterogeneity between 2 statins in the high−intensity trials analysis: χ^2_1 =0.3, p=0.57

Webfigure 1d: Effect of statin vs placebo on NEW−ONSET DIABETES, subdivided by statin intensity and trial

Trial	Dose	Events (% per annum): Statin (n=49114)	Events (% per annum): Placebo (n=49125)	Observed−Expected: O−E	Observed−Expected: Var(O−E)	Rate Ratio (95% or 99% CI)
**Low/moderate−intensity statin**						
**Lovastatin**						
AFCAPS/TexCAPS	20−40mg	175 (1.1)	136 (0.9)	19.0	77.7	1.28 (0.95 − 1.71)
**Subtotal**		**175 (1.1)**	**136 (0.9)**	**19.0**	**77.7**	**1.28 (1.02 − 1.59)**
**Fluvastatin**						
ALERT	40−80mg	30 (0.7)	35 (0.8)	−2.3	16.2	0.87 (0.46 − 1.65)
LIPS	80mg	4 (0.1)	10 (0.4)	−2.9	3.5	0.44 (0.11 − 1.72)
**Subtotal**		**34 (0.5)**	**45 (0.6)**	**−5.2**	**19.7**	**0.77 (0.49 − 1.20)**
**Simvastatin**						
4S	20−40mg	51 (0.5)	45 (0.4)	2.8	24.0	1.13 (0.67 − 1.90)
HPS	40mg	336 (0.9)	292 (0.8)	21.1	156.0	1.15 (0.93 − 1.41)
**Subtotal**		**387 (0.8)**	**337 (0.7)**	**24.0**	**180.0**	**1.14 (0.99 − 1.32)**
**Pravastatin**						
WOSCOPS	40mg	113 (0.7)	136 (0.9)	−12.4	62.2	0.82 (0.59 − 1.14)
CARE	40mg	196 (2.4)	166 (2.1)	13.4	90.4	1.16 (0.88 − 1.52)
LIPID	40mg	229 (1.0)	221 (1.0)	1.8	112.5	1.02 (0.80 − 1.30)
PROSPER	40mg	166 (2.1)	120 (1.5)	22.8	71.4	1.38 (1.01 − 1.87)
**Subtotal**		**704 (1.3)**	**643 (1.2)**	**25.6**	**336.6**	**1.08 (0.97 − 1.20)**
**Atorvastatin**						
ASCOT−LLA	10mg	359 (3.1)	328 (2.8)	14.2	171.6	1.09 (0.89 − 1.32)
**Subtotal**		**359 (3.1)**	**328 (2.8)**	**14.2**	**171.6**	**1.09 (0.94 − 1.26)**
**Rosuvastatin**						
CORONA	10mg	142 (3.3)	118 (2.8)	12.0	65.0	1.20 (0.87 − 1.66)
GISSI−HF	10mg	303 (7.0)	299 (6.5)	11.7	150.1	1.08 (0.88 − 1.33)
AURORA	10mg	74 (2.5)	83 (2.6)	−2.5	39.2	0.94 (0.62 − 1.41)
HOPE−3	10mg	242 (0.8)	225 (0.7)	8.1	116.7	1.07 (0.84 − 1.36)
**Subtotal**		**761 (1.8)**	**725 (1.7)**	**29.3**	**371.1**	**1.08 (0.98 − 1.20)**
**Any low/moderate−intensity**		**2420 (1.3)**	**2214 (1.2)**	**106.8**	**1156.8**	**1.10 (1.04 − 1.16)**
**High−intensity statin**						
**Atorvastatin**						
SPARCL	80mg	146 (1.7)	113 (1.3)	17.4	64.7	1.31 (0.95 − 1.80)
**Subtotal**		**146 (1.7)**	**113 (1.3)**	**17.4**	**64.7**	**1.31 (1.03 − 1.67)**
**Rosuvastatin**						
JUPITER	20mg	1075 (6.3)	792 (4.7)	146.4	466.0	1.37 (1.22 − 1.54)
**Subtotal**		**1075 (6.3)**	**792 (4.7)**	**146.4**	**466.0**	**1.37 (1.25 − 1.50)**
**Any high−intensity**		**1221 (4.8)**	**905 (3.5)**	**163.9**	**530.8**	**1.36 (1.25 − 1.48)**

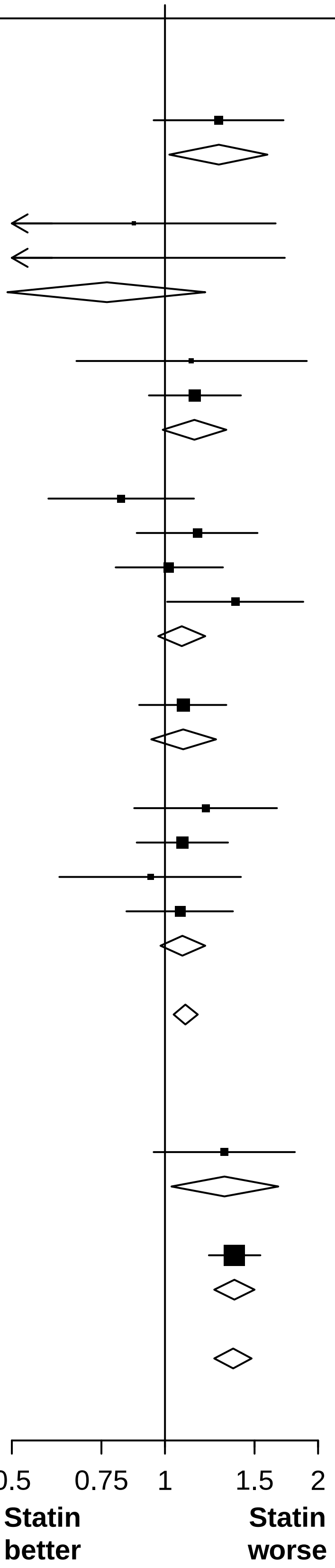

■ 99% or ◇ 95% CI

Test for heterogeneity between 6 statins in the low/moderate−intensity analysis: χ^2_5 =4.7, p=0.45

Test for heterogeneity between 2 statins in the high−intensity trials analysis: χ^2_1 =0.1, p=0.74

Webfigure 2: Effect of high−intensity statin vs placebo on NEW−ONSET DIABETES using different diagnosis criteria

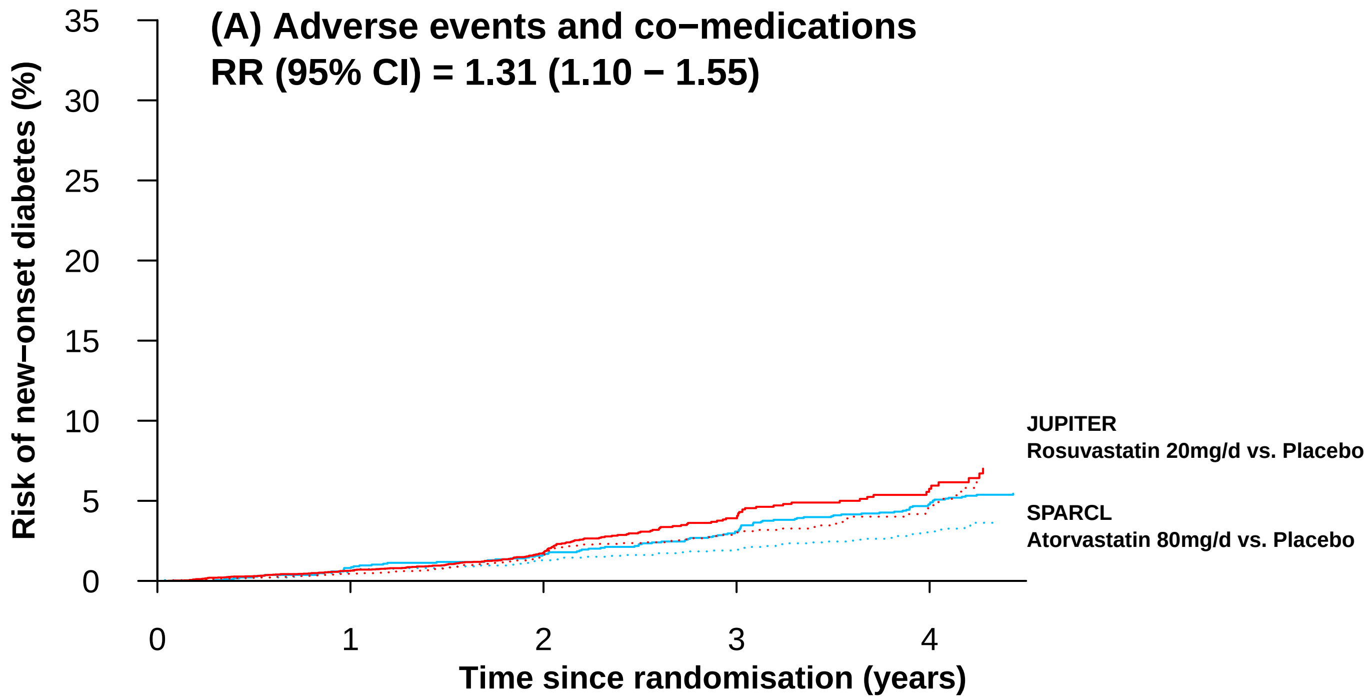

Number at risk

**JUPITER**					
**Rosuvatatin**	8026	7812	3655	1231	486
**Placebo**	7946	7748	3658	1263	511
**SPARCL**					
**Atorvastatin**	1909	1834	1780	1719	1647
**Placebo**	1913	1853	1810	1746	1689

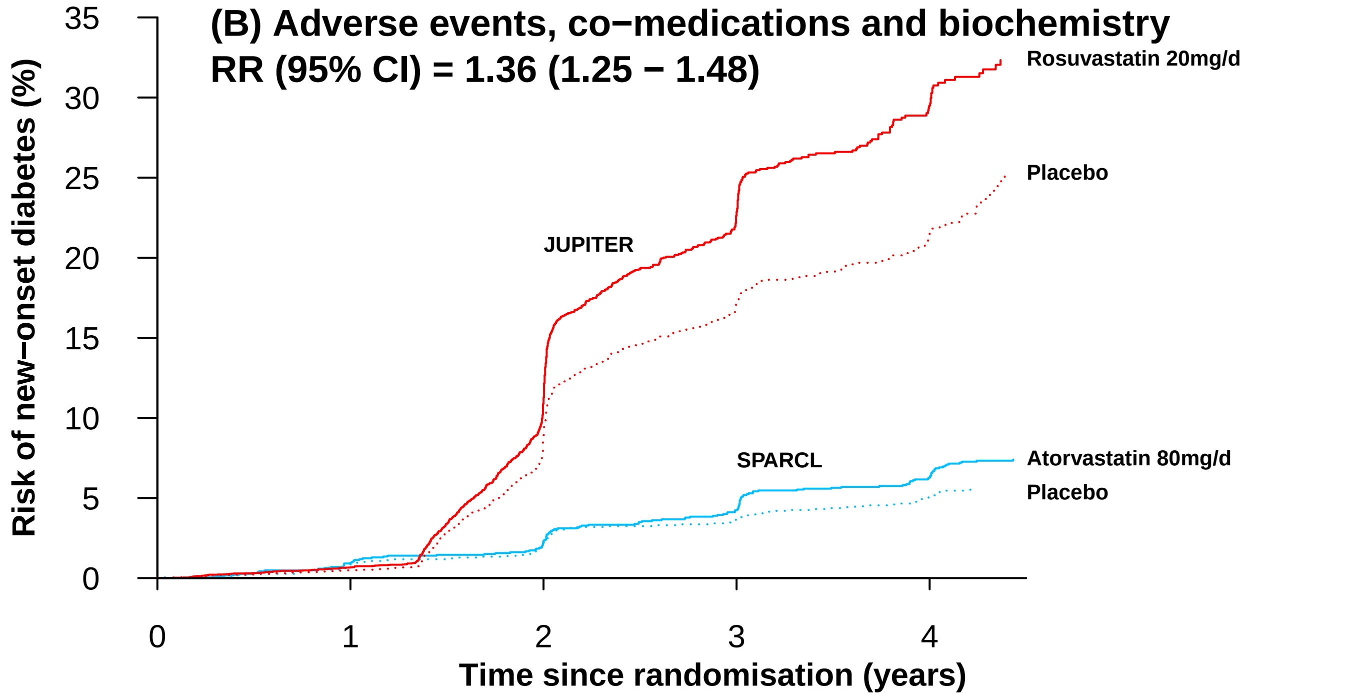

Number at risk

**JUPITER**					
**Rosuvatatin**	8026	7810	3592	1180	453
**Placebo**	7946	7747	3611	1235	495
**SPARCL**					
**Atorvastatin**	1909	1832	1771	1700	1622
**Placebo**	1913	1850	1794	1719	1656

Kaplan−Meier plots are shown to 4.5 years but all follow−up is used when calculating the overall RRs

Webfigure 3: Effect of more vs less intensive statin on NEW−ONSET DIABETES, subdivided by statin intensity and trial

Trial	Dose	Events (% per annum): More Statin (n=12726)	Events (% per annum): Less Statin (n=12658)	Observed−Expected: O−E	Observed−Expected: Var(O−E)	Rate Ratio (95% or 99% CI)
**Comparison of moderate−intensity regimens**						
A to Z	S40−80 vs S20	221 (7.6)	201 (7.2)	5.8	105.4	1.06 (0.82 − 1.36)
SEARCH	S80 vs S20	628 (1.8)	590 (1.7)	19.1	304.5	1.06 (0.92 − 1.23)
**Subtotal**		**849 (2.3)**	**791 (2.1)**	**24.8**	**409.9**	**1.06 (0.96 − 1.17)**
**Comparison of high vs moderate−intensity regimens**						
TNT	A80 vs A10	427 (2.2)	390 (2.0)	19.4	204.2	1.10 (0.92 − 1.32)
PROVE−IT	A80 vs P40	186 (6.4)	146 (4.9)	22.0	82.8	1.30 (0.98 − 1.73)
**Subtotal**		**613 (2.8)**	**536 (2.4)**	**41.4**	**287.0**	**1.16 (1.03 − 1.30)**
**All trials**		**1462 (2.5)**	**1327 (2.2)**	**66.2**	**696.8**	**1.10 (1.02 − 1.18)**

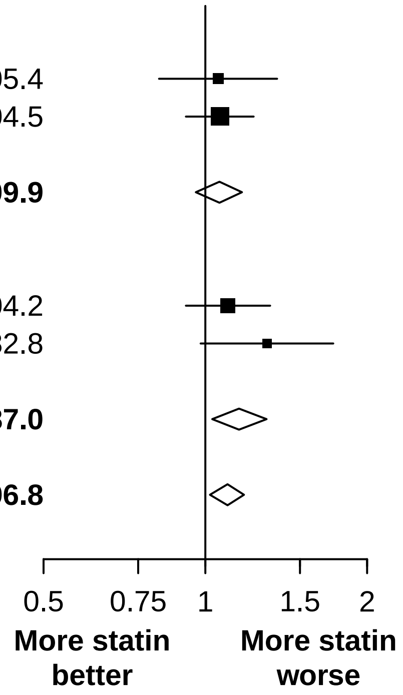

■ 99% or ◇ 95% CI

Webfigure 4: Effect of more vs less intensive statin on NEW−ONSET DIABETES and WORSENING GLYCAEMIA

	Events (% per annum)		Observed−Expected		
**Endpoint**	**More statin**	**Less statin**	**O−E**	**Var(O−E)**	**Rate Ratio (95% or 99% CI)**
**New−onset diabetes**	**(n=12726)**	**(n=12658)**			
Adverse event	887 (1.5)	799 (1.3)	44.2	421.5	1.11 (0.98 − 1.26)
Determined from co−medication	700 (1.2)	625 (1.0)	37.2	331.0	1.12 (0.97 − 1.29)
**Subtotal: AEs and comeds**	**998 (1.7)**	**881 (1.5)**	**58.5**	**469.6**	**1.13 (1.03 − 1.24)**
Biochemically determined	720 (1.2)	624 (1.0)	46.1	335.7	1.15 (1.00 − 1.32)
**Any new−onset diabetes**	**1462 (2.5)**	**1327 (2.2)**	**66.2**	**696.8**	**1.10 (1.02 − 1.18)**
**Worsening glycaemia**	**(n=2664)**	**(n=2676)**			
Ketosis/glucose control complications	49 (0.5)	56 (0.5)	−3.2	26.2	0.88 (0.53 − 1.46)
Worsening HbA1c	768 (8.7)	701 (7.7)	49.3	364.2	1.14 (1.00 − 1.31)
Escalation of diabetes co−medication	1179 (16.5)	1190 (16.2)	8.2	582.1	1.01 (0.91 − 1.13)
**Any worsening glycaemia**	**1515 (24.9)**	**1496 (23.6)**	**46.1**	**742.5**	**1.06 (0.99 − 1.14)**

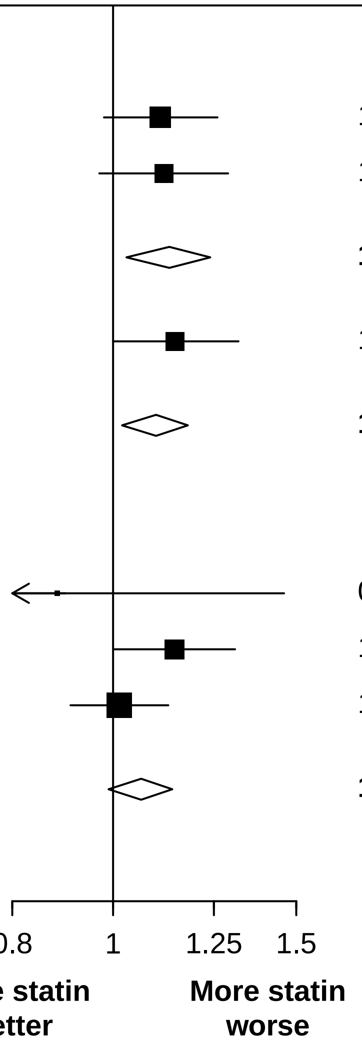

99% or 95% CI

See main figure legend for definitions

Webfigure 5: Effect of low/moderate−intensity statin vs placebo on NEW−ONSET DIABETES, subdivided by participants' characteristics

Subgroup	Events (% per annum) Statin (n=39179)	Events (% per annum) Placebo (n=39266)	Observed−Expected O−E	Observed−Expected Var(O−E)	Rate Ratio (95% or 99% CI)
**Age, years** (Trend: χ^2_1 =1.6, p=0.20)					
≤65	1356 (1.2)	1257 (1.2)	44.7	652.0	1.07 (0.97 − 1.18)
>65, ≤75	807 (1.4)	734 (1.3)	39.9	384.7	1.11 (0.97 − 1.26)
>75	254 (2.3)	220 (2.0)	23.9	118.0	1.22 (0.97 − 1.55)
**Sex** (Heterogeneity: χ^2_1 =1.4, p=0.23)					
Female	542 (1.2)	462 (1.1)	40.0	250.5	1.17 (1.00 − 1.38)
Male	1878 (1.4)	1752 (1.3)	67.4	906.0	1.08 (0.99 − 1.17)
**Race/Ethnicity** (Heterogeneity: χ^2_3 =9.3, p=0.03)					
Asian	104 (0.7)	122 (0.8)	−9.1	56.2	0.85 (0.60 − 1.20)
Black	42 (2.6)	34 (2.1)	2.7	18.7	1.16 (0.64 − 2.10)
Caucasian	1373 (1.6)	1252 (1.4)	65.4	654.8	1.11 (1.00 − 1.22)
Other	126 (1.1)	83 (0.7)	22.0	52.2	1.52 (1.07 − 2.18)
Missing	775 (1.2)	723 (1.1)	24.6	374.3	1.07 (0.93 − 1.22)
**History of vascular disease** (Heterogeneity: χ^2_1 =0.1, p=0.71)					
No	974 (1.2)	896 (1.1)	36.9	467.2	1.08 (0.96 − 1.22)
Yes	1446 (1.5)	1318 (1.3)	69.6	689.3	1.11 (1.00 − 1.22)
**Body mass index, kg/m^2** (Trend: χ^2_1 =4.9, p=0.03)					
≤25	414 (0.7)	455 (0.7)	−13.6	216.9	0.94 (0.79 − 1.12)
>25, ≤30	1114 (1.3)	987 (1.2)	56.5	524.1	1.11 (1.00 − 1.25)
>30	884 (2.7)	770 (2.4)	57.4	411.2	1.15 (1.01 − 1.31)
**Estimated GFR, mL/min/1.73m^2** (Trend: χ^2_1 =0.5, p=0.46)					
On dialysis	74 (2.5)	83 (2.6)	−2.5	39.2	0.94 (0.62 − 1.41)
≤60	709 (1.7)	626 (1.4)	48.4	332.9	1.16 (1.00 − 1.33)
>60, ≤90	1220 (1.3)	1115 (1.2)	52.2	582.7	1.09 (0.98 − 1.22)
>90	235 (1.0)	229 (1.0)	−0.4	115.6	1.00 (0.78 − 1.27)
**Quartile of glycaemia** (Heterogeneity: χ^2_3 =0.6, p=0.89)					
Quartile 1	236 (0.6)	230 (0.6)	1.1	116.3	1.01 (0.79 − 1.28)
Quartile 2	220 (0.7)	205 (0.6)	8.7	105.6	1.09 (0.84 − 1.39)
Quartile 3	387 (1.4)	359 (1.3)	18.5	185.7	1.10 (0.91 − 1.33)
Quartile 4	979 (4.0)	916 (3.8)	26.6	472.5	1.06 (0.94 − 1.19)
Missing	598 (1.1)	504 (0.9)	43.8	274.2	1.17 (1.00 − 1.37)
**Quartile of diabetes risk score** (Heterogeneity: χ^2_3 =0.3, p=0.96)					
Quartile 1	288 (0.4)	257 (0.3)	16.6	136.2	1.13 (0.91 − 1.41)
Quartile 2	407 (0.8)	378 (0.8)	13.1	195.7	1.07 (0.89 − 1.29)
Quartile 3	592 (2.0)	542 (1.9)	22.2	281.7	1.08 (0.93 − 1.26)
Quartile 4	1133 (6.0)	1037 (5.5)	50.2	540.5	1.10 (0.98 − 1.23)
**Total**	**2420 (1.3)**	**2214 (1.2)**	**106.8**	**1156.8**	**1.10 (1.04 − 1.16)**

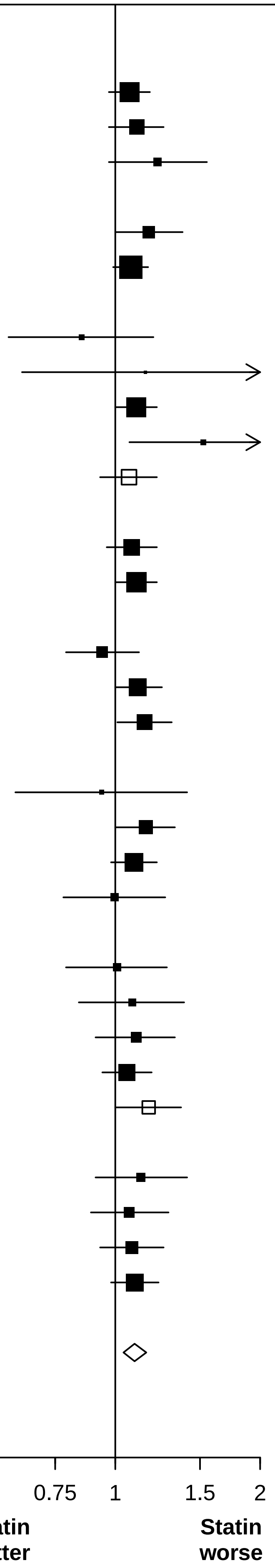

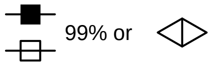

Webfigure 6: Effect of low/moderate−intensity statin vs placebo on NEW−ONSET DIABETES, subdivided by participants' lipid characteristics

Subgroup	Events (% per annum): Statin (n=39179)	Events (% per annum): Placebo (n=39266)	Observed−Expected: O−E	Observed−Expected: Var(O−E)	Rate Ratio (95% or 99% CI)
**LDL−C, mmol/L** (Trend: χ^2_1 =3.0, p=0.08)					
<2.0	91 (1.9)	87 (1.8)	0.5	44.0	1.01 (0.69 − 1.49)
≥ 2.0, <2.5	180 (1.9)	137 (1.4)	25.4	78.7	1.38 (1.03 − 1.85)
≥ 2.5, <3.0	313 (1.6)	261 (1.3)	30.9	142.4	1.24 (1.00 − 1.54)
≥ 3.0, <3.5	503 (1.5)	464 (1.4)	19.2	241.2	1.08 (0.92 − 1.28)
≥ 3.5	1231 (1.1)	1157 (1.1)	34.8	596.1	1.06 (0.95 − 1.18)
**HDL−C, mmol/L** (Trend: χ^2_1 =0.7, p=0.41)					
≤1.0	1040 (1.7)	980 (1.6)	26.3	503.4	1.05 (0.94 − 1.18)
>1.0, ≤ 1.3	784 (1.3)	703 (1.1)	48.5	370.9	1.14 (1.00 − 1.30)
> 1.3	419 (1.2)	380 (1.0)	19.9	199.5	1.10 (0.92 − 1.33)
**Triglycerides, mmol/L** (Trend: χ^2_1 =1.4, p=0.24)					
≤1.4	730 (1.1)	662 (1.0)	41.6	347.5	1.13 (0.98 − 1.29)
>1.4, ≤ 2.0	614 (1.3)	548 (1.2)	35.7	289.9	1.13 (0.97 − 1.32)
>2.0	998 (1.7)	936 (1.7)	19.9	481.7	1.04 (0.93 − 1.17)
**Total**	**2420 (1.3)**	**2214 (1.2)**	**106.8**	**1156.8**	**1.10 (1.04 − 1.16)**

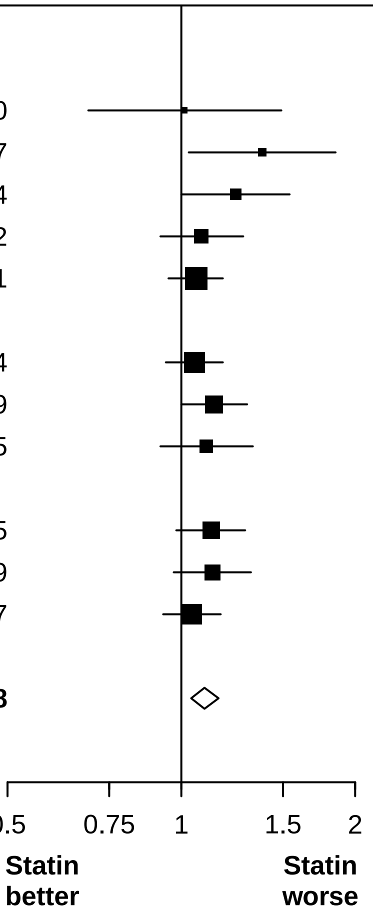

■ 99% or ◇ 95% CI

Webfigure 7: Effect of high−intensity statin vs placebo on NEW−ONSET DIABETES, subdivided by participants' characteristics

Subgroup	Events (% per annum): Statin (n=9935)	Events (% per annum): Placebo (n=9859)	Observed−Expected: O−E	Observed−Expected: Var(O−E)	Rate Ratio (95% or 99% CI)
**Age, years** (Trend: χ^2_1 =0.1, p=0.97)					
≤65	564 (4.4)	440 (3.3)	71.8	250.6	1.33 (1.13 − 1.57)
>65, ≤75	537 (5.5)	362 (3.9)	79.7	224.4	1.43 (1.20 − 1.69)
>75	120 (3.8)	103 (3.2)	11.4	55.6	1.23 (0.87 − 1.73)
**Sex** (Heterogeneity: χ^2_1 =0.1, p=0.80)					
Female	482 (5.0)	348 (3.5)	66.6	207.1	1.38 (1.15 − 1.65)
Male	739 (4.6)	557 (3.5)	96.6	323.5	1.35 (1.17 − 1.56)
**Race/Ethnicity** (Heterogeneity: χ^2_3 =0.4, p=0.95)					
Asian	2 (3.5)	2 (4.0)	−0.1	1.0	0.87 (0.07 − 11.49)
Black	6 (3.0)	5 (2.6)	1.0	2.5	1.50 (0.29 − 7.64)
Caucasian	133 (1.6)	103 (1.2)	15.9	59.0	1.31 (0.94 − 1.83)
Other	5 (2.4)	3 (1.4)	1.1	2.0	1.73 (0.28 − 10.70)
Missing	1075 (6.3)	792 (4.7)	146.4	466.0	1.37 (1.22 − 1.54)
**History of vascular disease** (Heterogeneity: χ^2_1 =0.1, p=0.74)					
No	1075 (6.3)	792 (4.7)	146.4	466.0	1.37 (1.22 − 1.54)
Yes	146 (1.7)	113 (1.3)	17.4	64.7	1.31 (0.95 − 1.80)
**Body mass index, kg/m^2** (Trend: χ^2_1 =5.0, p=0.02)					
≤25	151 (3.3)	121 (2.7)	13.1	67.9	1.21 (0.89 − 1.66)
>25, ≤30	358 (4.6)	292 (3.8)	32.8	162.3	1.22 (1.00 − 1.50)
>30	597 (8.9)	407 (5.9)	106.3	250.3	1.53 (1.30 − 1.80)
**Estimated GFR, mL/min/1.73m^2** (Trend: χ^2_1 =0.8, p=0.37)					
On dialysis	−	−			
≤60	284 (4.1)	213 (3.1)	39.7	124.0	1.38 (1.09 − 1.74)
>60, ≤90	804 (4.9)	566 (3.4)	115.8	342.0	1.40 (1.22 − 1.61)
>90	132 (5.8)	126 (5.2)	9.0	64.1	1.15 (0.83 − 1.59)
**Quartile of glycaemia** (Heterogeneity: χ^2_3 =1.7, p=0.63)					
Quartile 1	51 (1.2)	43 (1.0)	2.1	23.4	1.10 (0.64 − 1.86)
Quartile 2	139 (1.8)	100 (1.3)	21.2	59.7	1.43 (1.02 − 1.99)
Quartile 3	323 (4.0)	223 (2.7)	51.5	136.3	1.46 (1.17 − 1.82)
Quartile 4	707 (13.2)	539 (9.8)	112.1	308.0	1.44 (1.24 − 1.67)
Missing	1 (1.0)	0 (0.0)	0.4	0.2	
**Quartile of diabetes risk score** (Heterogeneity: χ^2_3 =2.3, p=0.52)					
Quartile 1	17 (0.5)	19 (0.5)	−1.3	9.0	0.87 (0.37 − 2.05)
Quartile 2	37 (1.0)	31 (0.8)	3.1	17.0	1.20 (0.64 − 2.24)
Quartile 3	123 (1.9)	87 (1.3)	18.5	52.5	1.42 (1.00 − 2.03)
Quartile 4	1044 (8.8)	768 (6.4)	147.0	451.7	1.38 (1.23 − 1.56)
**Total**	**1221 (4.8)**	**905 (3.5)**	**163.9**	**530.8**	**1.36 (1.25 − 1.48)**

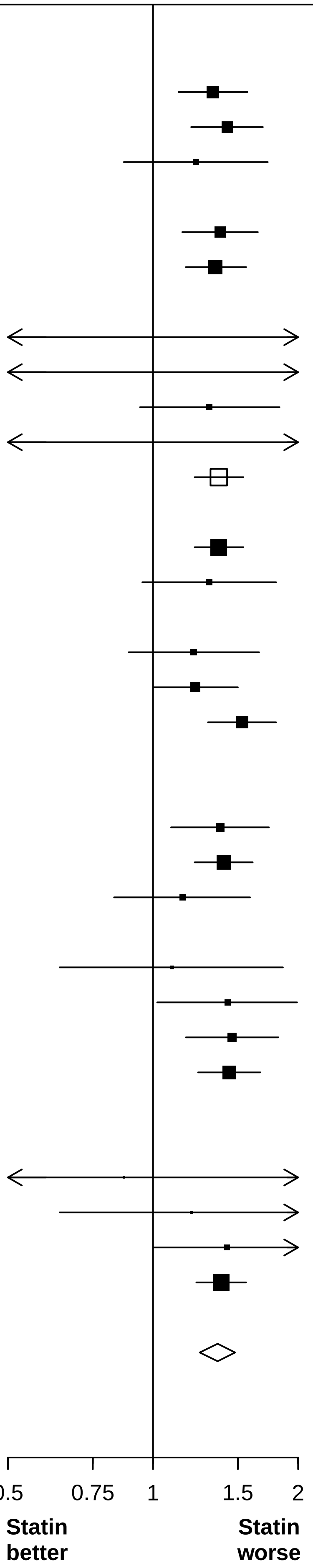

99% or 95% CI

Webfigure 8: Effect of high−intensity statin vs placebo on NEW−ONSET DIABETES, subdivided by participants' lipid characteristics

Subgroup	Events (% per annum): Statin (n=9935)	Events (% per annum): Placebo (n=9859)	Observed−Expected: O−E	Observed−Expected: Var(O−E)	Rate Ratio (95% or 99% CI)
**LDL−C, mmol/L** (Trend: χ^2_1 =0.1, p=0.95)					
<2.0	116 (7.4)	86 (6.0)	11.3	50.2	1.25 (0.87 − 1.80)
≥ 2.0, <2.5	193 (5.5)	147 (4.1)	26.4	84.8	1.37 (1.03 − 1.81)
≥ 2.5, <3.0	428 (5.2)	304 (3.7)	62.4	182.5	1.41 (1.16 − 1.70)
≥ 3.0, <3.5	423 (5.0)	313 (3.6)	58.6	183.7	1.38 (1.14 − 1.66)
≥ 3.5	58 (1.5)	50 (1.3)	4.0	27.0	1.16 (0.71 − 1.91)
**HDL−C, mmol/L** (Trend: χ^2_1 =0.1, p=0.87)					
≤1.0	313 (6.5)	227 (4.7)	43.8	134.8	1.38 (1.11 − 1.73)
>1.0, ≤ 1.3	470 (5.1)	359 (3.9)	56.1	206.9	1.31 (1.10 − 1.57)
> 1.3	437 (3.8)	319 (2.7)	63.2	188.6	1.40 (1.16 − 1.69)
**Triglycerides, mmol/L** (Trend: χ^2_1 =0.2, p=0.65)					
≤1.4	508 (3.8)	402 (2.9)	61.4	227.1	1.31 (1.10 − 1.55)
>1.4, ≤ 2.0	336 (5.0)	233 (3.6)	49.2	142.0	1.41 (1.14 − 1.76)
>2.0	376 (6.7)	270 (4.8)	50.3	161.1	1.37 (1.12 − 1.67)
**Total**	**1221 (4.8)**	**905 (3.5)**	**163.9**	**530.8**	**1.36 (1.25 − 1.48)**

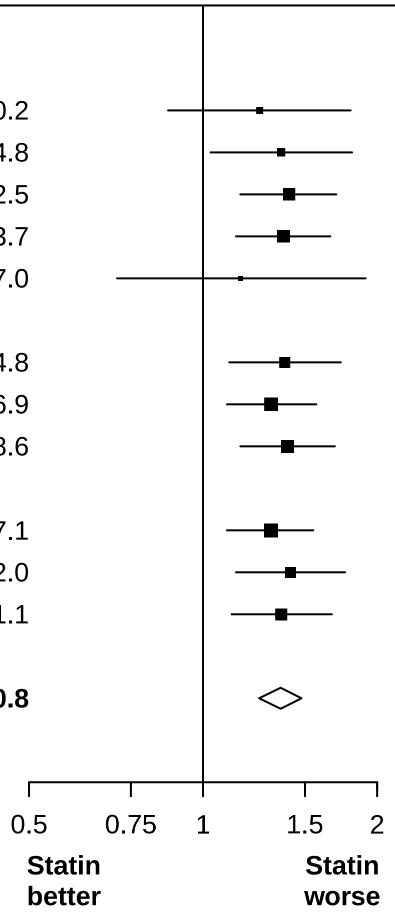

■ 99% or ◇ 95% CI

Webfigure 9: Effect of more vs less intensive statin on NEW−ONSET DIABETES, subdivided by participants' characteristics

Subgroup	Events (% per annum): More Statin (n=12726)	Events (% per annum): Less Statin (n=12658)	Observed−Expected: O−E	Observed−Expected: Var(O−E)	Rate Ratio (95% or 99% CI)
**Age, years** (Trend: χ^2_1 =1.9, p=0.17)					
≤65	947 (2.7)	823 (2.3)	60.1	442.1	1.15 (1.01 − 1.30)
>65, ≤75	449 (2.2)	433 (2.2)	7.2	220.3	1.03 (0.87 − 1.23)
>75	65 (2.0)	71 (2.1)	−0.4	33.9	0.99 (0.63 − 1.54)
**Sex** (Heterogeneity: χ^2_1 =1.0, p=0.32)					
Female	259 (2.5)	255 (2.5)	2.0	128.4	1.02 (0.81 − 1.28)
Male	1203 (2.5)	1072 (2.2)	64.1	568.3	1.12 (1.00 − 1.25)
**Race/Ethnicity** (Heterogeneity: χ^2_3 =4.2, p=0.24)					
Asian	32 (4.7)	22 (3.2)	5.3	13.4	1.49 (0.74 − 3.00)
Black	37 (6.4)	31 (4.4)	7.3	16.3	1.56 (0.83 − 2.95)
Caucasian	1359 (2.4)	1252 (2.2)	50.6	652.3	1.08 (0.98 − 1.20)
Other	34 (5.3)	22 (3.9)	4.7	13.9	1.40 (0.70 − 2.80)
Missing	−	−			
**History of vascular disease**					
No	−	−			
Yes	1462 (2.5)	1327 (2.2)	66.2	696.8	1.10 (1.00 − 1.21)
**Body mass index, kg/m^2** (Trend: χ^2_1 =0.1, p=0.94)					
≤25	243 (1.6)	211 (1.4)	16.1	113.4	1.15 (0.90 − 1.47)
>25, ≤30	570 (2.2)	536 (2.1)	16.7	276.2	1.06 (0.91 − 1.24)
>30	629 (3.7)	553 (3.2)	31.7	295.0	1.11 (0.96 − 1.29)
**Estimated GFR, mL/min/1.73m^2** (Trend: χ^2_1 =0.1, p=0.89)					
On dialysis	−	−			
≤60	332 (2.7)	304 (2.5)	13.9	158.8	1.09 (0.89 − 1.34)
>60, ≤90	843 (2.3)	758 (2.1)	37.4	399.8	1.10 (0.97 − 1.25)
>90	248 (2.5)	228 (2.1)	12.4	118.2	1.11 (0.88 − 1.41)
**Quartile of glycaemia** (Heterogeneity: χ^2_3 =0.6, p=0.89)					
Quartile 1	74 (1.5)	73 (1.5)	1.1	36.7	1.03 (0.67 − 1.58)
Quartile 2	83 (1.5)	80 (1.4)	2.8	40.5	1.07 (0.72 − 1.61)
Quartile 3	134 (2.0)	118 (1.9)	5.3	62.6	1.09 (0.79 − 1.51)
Quartile 4	498 (7.0)	430 (6.0)	34.0	231.2	1.16 (0.98 − 1.37)
Missing	673 (1.9)	626 (1.8)	24.0	324.7	1.08 (0.93 − 1.24)
**Quartile of diabetes risk score** (Heterogeneity: χ^2_3 =1.2, p=0.74)					
Quartile 1	35 (0.7)	28 (0.5)	3.3	15.7	1.23 (0.64 − 2.36)
Quartile 2	106 (0.7)	101 (0.7)	4.0	51.7	1.08 (0.75 − 1.54)
Quartile 3	471 (1.9)	444 (1.8)	9.5	228.7	1.04 (0.88 − 1.24)
Quartile 4	850 (6.3)	754 (5.5)	50.2	400.4	1.13 (1.00 − 1.29)
**Total**	**1462 (2.5)**	**1327 (2.2)**	**66.2**	**696.8**	**1.10 (1.02 − 1.18)**

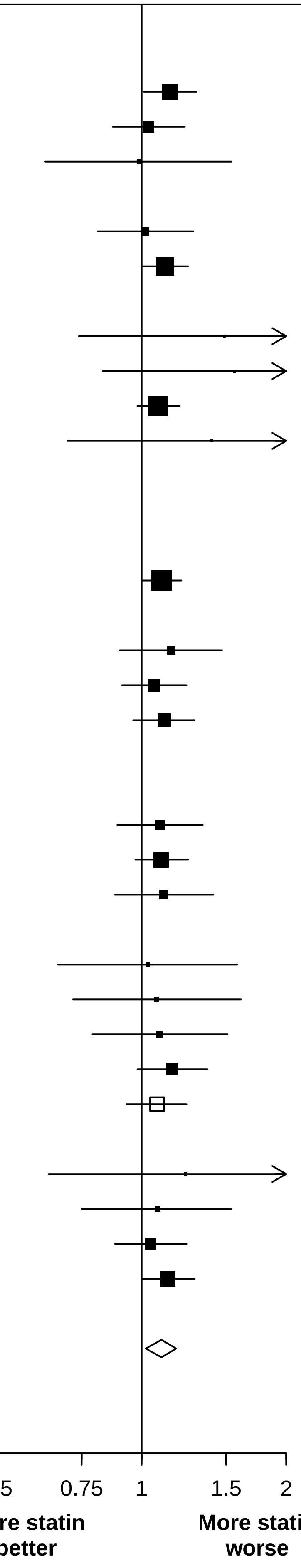

99% or 95% CI

Webfigure 10: Effect of more vs less intensive statin on NEW−ONSET DIABETES, subdivided by participants' lipid characteristics

Subgroup	Events (% per annum): More Statin (n=12726)	Events (% per annum): Less Statin (n=12658)	Observed−Expected: O−E	Observed−Expected: Var(O−E)	Rate Ratio (95% or 99% CI)
**LDL−C, mmol/L** (Trend: χ^2_1 =0.2, p=0.66)					
<2.0	284 (2.7)	269 (2.5)	10.9	138.0	1.08 (0.87 − 1.35)
≥ 2.0, <2.5	457 (2.3)	401 (2.1)	18.7	214.3	1.09 (0.92 − 1.30)
≥ 2.5, <3.0	433 (2.6)	386 (2.2)	29.9	204.3	1.16 (0.97 − 1.39)
≥ 3.0, <3.5	186 (2.3)	183 (2.3)	1.9	92.1	1.02 (0.78 − 1.34)
≥ 3.5	85 (2.6)	65 (2.0)	9.1	37.4	1.27 (0.84 − 1.94)
**HDL−C, mmol/L** (Trend: χ^2_1 =0.5, p=0.48)					
≤1.0	651 (2.9)	597 (2.7)	29.3	311.8	1.10 (0.95 − 1.27)
>1.0, ≤ 1.3	458 (2.2)	430 (2.1)	12.2	221.7	1.06 (0.89 − 1.26)
> 1.3	205 (1.4)	162 (1.1)	20.3	91.7	1.25 (0.95 − 1.63)
**Triglycerides, mmol/L** (Trend: χ^2_1 =0.3, p=0.56)					
≤1.4	252 (1.2)	233 (1.1)	9.8	121.2	1.08 (0.86 − 1.37)
>1.4, ≤ 2.0	362 (2.3)	335 (2.1)	11.0	174.0	1.07 (0.88 − 1.29)
>2.0	821 (3.9)	729 (3.4)	48.5	387.1	1.13 (0.99 − 1.29)
**Total**	**1462 (2.5)**	**1327 (2.2)**	**66.2**	**696.8**	**1.10 (1.02 − 1.18)**

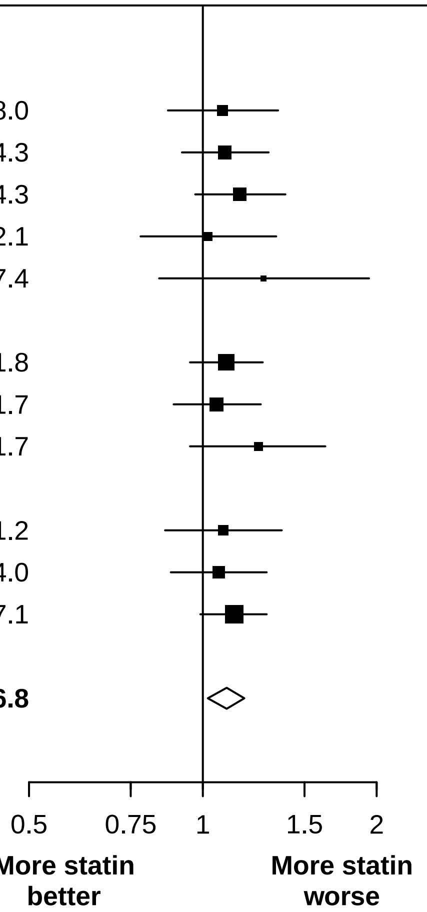

■ 99% or ◇ 95% CI

Webfigure 11: Effect of statin vs placebo on NEW−ONSET DIABETES, subdivided by duration of treatment and statin intensity

	Events (% per annum)		Observed−Expected		
**Year**	**Statin**	**Placebo**	**O−E**	**Var(O−E)**	**Rate Ratio (95% or 99% CI)**
**Low/moderate−intensity statin**	**(n=39179)**	**(n=39266)**			
Year 1	439 (1.1)	417 (1.1)	13.7	213.8	1.07 (0.89 − 1.27)
Year 2	489 (1.3)	434 (1.2)	30.3	230.6	1.14 (0.96 − 1.35)
Year 3	488 (1.4)	485 (1.4)	2.7	243.1	1.01 (0.86 − 1.19)
Year 4	402 (1.3)	363 (1.2)	19.7	191.2	1.11 (0.92 − 1.34)
Years 5+	602 (1.6)	515 (1.4)	40.5	278.2	1.16 (0.99 − 1.35)
**All years**	**2420 (1.3)**	**2214 (1.2)**	**106.8**	**1156.8**	**1.10 (1.04 − 1.16)**
**High−intensity statin**	**(n=9935)**	**(n=9859)**			
Year 1	71 (0.7)	54 (0.6)	8.4	31.2	1.31 (0.82 − 2.07)
Year 2	607 (7.6)	481 (6.1)	62.1	271.8	1.26 (1.07 − 1.47)
Year 3	373 (10.2)	261 (7.0)	59.2	158.2	1.45 (1.18 − 1.78)
Year 4	118 (4.8)	69 (2.7)	26.9	46.7	1.78 (1.22 − 2.59)
Years 5+	52 (2.8)	40 (2.1)	7.2	22.9	1.37 (0.80 − 2.35)
**All years**	**1221 (4.8)**	**905 (3.5)**	**163.9**	**530.8**	**1.36 (1.25 − 1.48)**

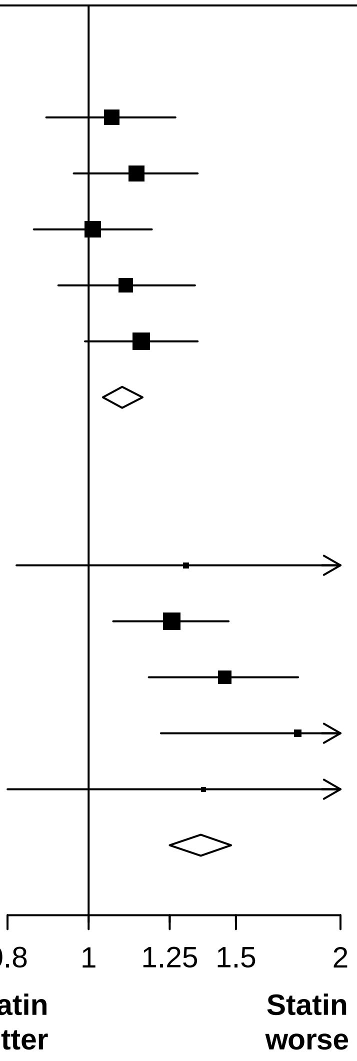

99% or 95% CI

For each risk period, percentages shown are of those alive and still at risk of a first report of incident of diabetes at the start of the risk period.

For low/moderate−intensity statin trials, the trend test for duration was: χ^2_1 =0.5, p=0.48

For high−intensity statin trials, the trend test for duration was: χ^2_1 =3.2, p=0.07

Webfigure 12: Effect of more vs less intensive statin on NEW−ONSET DIABETES and WORSENING GLYCAEMIA, subdivided by duration of treatment

	Events (% per annum)		Observed−Expected		
**Year**	**More statin**	**Less statin**	**O−E**	**Var(O−E)**	**Rate Ratio (95% or 99% CI)**
**New−onset diabetes**	**(n=12726)**	**(n=12658)**			
Year 1	354 (2.9)	273 (2.2)	38.8	156.6	1.28 (1.04 − 1.57)
Year 2	300 (2.6)	283 (2.5)	7.7	145.7	1.05 (0.85 − 1.31)
Year 3	214 (2.3)	226 (2.5)	−5.7	109.9	0.95 (0.74 − 1.21)
Year 4	189 (2.2)	158 (1.9)	15.7	86.7	1.20 (0.91 − 1.58)
Years 5+	405 (2.3)	387 (2.2)	9.8	198.0	1.05 (0.88 − 1.26)
**All years**	**1462 (2.5)**	**1327 (2.2)**	**66.2**	**696.8**	**1.10 (1.02 − 1.18)**
**Worsening glycaemia**	**(n=2664)**	**(n=2676)**			
Year 1	702 (30.8)	649 (27.9)	33.3	331.4	1.11 (0.96 − 1.27)
Year 2	409 (27.7)	369 (23.5)	33.7	192.8	1.19 (0.99 − 1.43)
Year 3	193 (23.8)	227 (26.0)	−6.7	103.2	0.94 (0.73 − 1.21)
Year 4	94 (15.9)	118 (18.7)	−7.6	52.7	0.87 (0.61 − 1.23)
Years 5+	117 (12.5)	133 (14.0)	−6.3	62.3	0.90 (0.65 − 1.25)
**All years**	**1515 (24.9)**	**1496 (23.6)**	**46.4**	**742.5**	**1.06 (0.99 − 1.14)**

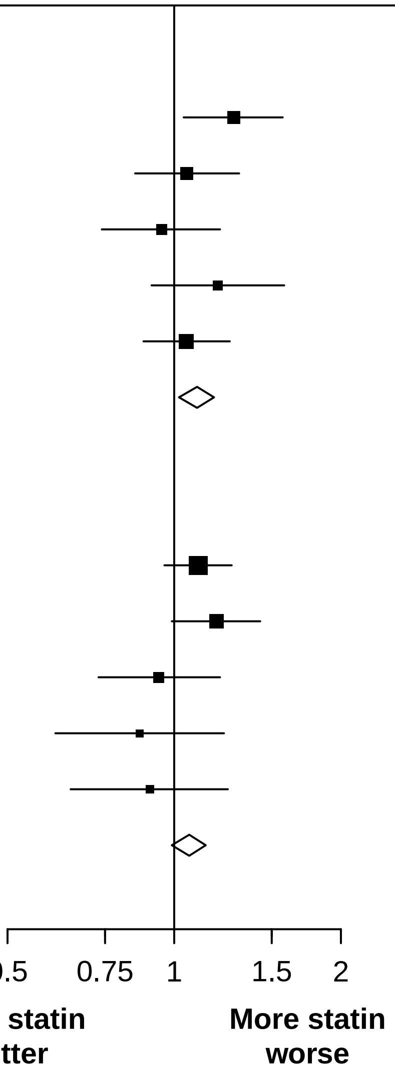

99% or 95% CI

For each risk period, percentages shown are of those alive and still at risk of a first report of incident of diabetes or worsening glycaemia at the start of the risk period.

For new−onset diabetes, the trend test for duration was: χ^2_1 =1.6, p=0.20

For worsening glycaemia, the trend test for duration was: χ^2_1 =5.1, p=0.02

Webfigure 13: Effect of statin vs placebo on WORSENING GLYCAEMIA, subdivided by duration of treatment and statin intensity

Year	Events (% per annum) Statin	Events (% per annum) Placebo	Observed−Expected O−E	Observed−Expected Var(O−E)	Rate Ratio (95% or 99% CI)
**Low/moderate−intensity statin**	**(n=12109)**	**(n=11941)**			
Year 1	2127 (19.0)	1855 (16.7)	155.4	980.0	1.17 (1.08 − 1.27)
Year 2	1164 (13.4)	1116 (12.6)	57.7	566.6	1.11 (0.99 − 1.23)
Year 3	704 (9.8)	693 (9.5)	27.8	347.4	1.08 (0.94 − 1.24)
Year 4	398 (7.2)	379 (6.8)	17.5	193.4	1.09 (0.91 − 1.32)
Years 5+	1831 (33.2)	1859 (33.9)	−6.4	650.2	0.99 (0.90 − 1.10)
**All years**	**6224 (16.3)**	**5902 (15.4)**	**252.0**	**2737.5**	**1.10 (1.06 − 1.14)**
**High−intensity statin**	**(n=805)**	**(n=846)**			
Year 1	75 (10.0)	72 (9.0)	3.2	36.7	1.09 (0.71 − 1.67)
Year 2	133 (23.1)	108 (17.5)	17.8	59.9	1.35 (0.97 − 1.88)
Year 3	66 (19.9)	57 (16.0)	6.0	30.5	1.22 (0.76 − 1.94)
Year 4	46 (17.6)	29 (10.0)	9.3	18.5	1.65 (0.91 − 3.00)
Years 5+	18 (9.3)	29 (12.0)	−3.1	11.5	0.77 (0.36 − 1.64)
**All years**	**338 (16.0)**	**295 (12.8)**	**33.3**	**157.2**	**1.24 (1.06 − 1.44)**

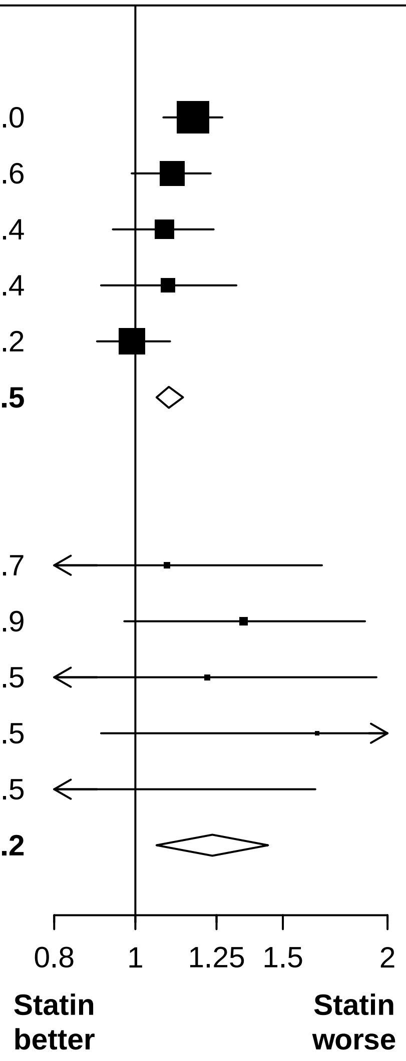

99% or 95% CI

For low/moderate−intensity statin trials, the trend test for duration was: χ^2_1 =10.5, p=0.001

For high−intensity statin trials, the trend test for duration was: χ^2_1 =0.1, p=0.91

Statistical Appendix

Stratifying participants into baseline risk groups of new-onset diabetes

Among the ~123,000 participants without prior diabetes in all 20 trials of a statin vs placebo or of a more intensive vs a less intensive statin regimen, a risk model was first developed to subdivide them into baseline risk groups. For participant i in trial j, let Y_{ij} denote the occurrence or otherwise for that patient of new onset diabetes during the trial, and let T_{ij} denote the number of years of follow-up. The logarithm of the expected annual event rate was modelled through the Poisson regression model:

$$ln\left(\frac{E(Y_{ij})}{T_{ij}}\right) = \alpha_j + (x_{ij} - \bar{x}_j)'\beta + w_{ij}'\gamma$$

where α_j is the average log annual event rate observed in trial j, x_{ij} is the vector of baseline characteristics for patient i in trial j, $\bar{x}_j$ is the vector of mean baseline risk characteristics observed in study j, β is the vector of regression coefficients associated with those baseline characteristics, w_{ij} reflects the treatment allocation for participant i in trial j (coded as placebo, low intensity statin or high intensity statin) and γ is the vector of regression coefficients associated with those treatment allocations. The baseline characteristics included were age (per year), sex, body mass index (per 1 kg/m^2), triglycerides (per 1 mmol/L), eGFR (per 1 mL/min/ 1.73m^2), HDL-C (per 1 mmol/L) and quartile of glycaemia (see Methods). Missing values were replaced with trial-specific averages (for continuous variables) or the modal category (for categorical variables), but where data were missing for all participants in a given trial (eg, eGFR in the 4S trial) a constant value was used (allowing information from that trial on other baseline characteristics to contribute to the model but without having any influence on the regression coefficient for the missing variable). In 3 trials, BMI was supplied as a categorical variable and so appropriate BMI values based on the cutpoints were used. The model also included a dispersion parameter (estimated by the deviance divided by the degrees of freedom).

For participant i in trial j, the predicted probability of new-onset diabetes within 5 years *had they been allocated placebo* was then estimated by:

$$P_{ij} = 100 \times (1 - (1 - e^{\eta_{ij}})^5)$$

where

$$\eta_{ij} = \hat{\alpha}_j + (x_{ij} - \bar{x}_j)'\hat{\beta}$$

Individuals were then categorised into four groups on the basis of the quartiles of P_{ij} (<2.9%, ≥2.9% to <5.7%, ≥5.7% to <11.5% and ≥11.5%). For the 3 trials (AFCAPS/TexCAPS, 4S and A to Z) where data were only available on a data sharing platform (rather than sent to the co-ordinating centre), P_{ij} was calculated by combining the regression coefficients $\hat{\beta}$ with the appropriate trial-specific average log annual event rate for the trial.
